# Model-based predictions of protective HIV pre-exposure prophylaxis adherence levels in cisgender women

**DOI:** 10.1038/s41591-023-02615-x

**Published:** 2023-11-13

**Authors:** Lanxin Zhang, Sara Iannuzzi, Ayyappa Chaturvedula, Elizabeth Irungu, Jessica E. Haberer, Craig W. Hendrix, Max von Kleist

**Affiliations:** 1 Project group 5 ‘Systems Medicine of Infectious Diseases’, Robert Koch Institute, Berlin, Germany; 2 International Max-Planck Research School ‘Biology and Computation’, Max-Planck Institute for Molecular Genetics, Berlin, Germany; 3 Department of Pharmacotherapy, University of North Texas Health Science Center, Fort Worth, TX USA; 4 Jhpiego Corporation, Nairobi, Kenya; 5 Center for Global Health, Massachusetts General Hospital, Boston, MA USA; 6 Department of Medicine, Harvard Medical School, Boston, MA USA; 7 Division of Clinical Pharmacology, Johns Hopkins University, Baltimore, MD USA; 8 Department of Mathematics and Computer Science, Freie Universität Berlin, Berlin, Germany

**Keywords:** HIV infections, Epidemiology

## Abstract

Most human immunodeficiency virus (HIV) infections occur in cisgender women in resource-limited settings. In women, self-protection with emtricitabine/tenofovir disoproxil fumarate pre-exposure prophylaxis (FTC/TDF-PrEP) constitutes a major pillar of HIV prevention. However, clinical trials in women had inconsistent outcomes, sparking uncertainty about adherence requirements and reluctance in evaluating on-demand regimens. We analyzed data from published FTC/TDF-PrEP trials to establish efficacy ranges in cisgender women. In a ‘bottom-up’ approach, we modeled hypotheses in the context of risk-group-specific, adherence–efficacy profiles and challenged those hypotheses with clinical data. We found that different clinical outcomes were related to the proportion of women taking the product, allowing coherent interpretation of the data. Our analysis showed that 90% protection was achieved when women took some product. We found that hypotheses of putative male/female differences were either not impactful or statistically inconsistent with clinical data. We propose that differing clinical outcomes could arise from pill-taking behavior rather than biological factors driving specific adherence requirements in cisgender women.

## Abstract (web summary)

Modeling shows that antiretroviral treatment as pre-exposure prophylaxis (PrEP) can be highly effective in cisgender women at preventing HIV-1 acquisition, but underscores the need to understand the barriers that limit PrEP adherence in women.

## Main

Most of the 1.5 million HIV infections in 2021 (ref. ^1^) occurred in sub-Saharan Africa, where young, cisgender women are disproportionally affected^2^. Consequently, sub-Saharan women remain a highly impacted risk group in need of options for HIV prevention. HIV PrEP is the most effective biomedical means, to date, by which women have control of protection against HIV acquisition. Although new long-acting PrEP regimens are becoming available^3,4^, generic PrEP with oral FTC/TDF is accessible in many resource-limited settings. However, uncertainties exist about adherence requirements for achieving HIV protection. As a result of these uncertainties, it is currently recommended that women take FTC/TDF-based PrEP once daily—an adherence requirement that may negatively affect PrEP uptake and persistence^5,6^.

Clinically, average PrEP efficacy is quantified based on relative incidence reductions (control versus PrEP intervention) across observational cohorts. However, as HIV transmission per sexual exposure is relatively low^7^, major limitations arise^8^: first, clinical estimates of average PrEP efficacy are statistically uncertain; second, they average over heterogeneous risk- and PrEP-adherence behavior; and, third, as large observation periods and cohorts are required, causative factors that influence per-exposure HIV risk reduction cannot be identified in these data.

In heterosexual women, the range in clinically estimated average efficacy of oral FTC/TDF-PrEP is particularly vast^9,10^. Specifically, some early studies pointed toward lower average risk reduction in heterosexual women, compared with men-who-have-sex-with-men (MSM)^9,11,12^. However, it is unclear, to date, whether this putatively lower efficacy is a consequence of intrinsic differences in physiology and drug pharmacokinetics at the virus exposure site or an artefact of poorly quantified and differing levels of adherence across studies, because many participants who acquired HIV in these trials may have simply not taken PrEP around the time of HIV exposure.

For developing PrEP guidelines, the existence of intrinsic differences is relevant, because it necessitates risk-group-specific recommendations on minimal adherence levels. The current World Health Organization guidelines for PrEP differentiate between heterosexual cisgender women and MSM^13^. Although PrEP on demand is considered safe in MSM based on the IPERGAY and PREVENIR studies^14^, no such study has been attempted in women.

**Fig. 1:**
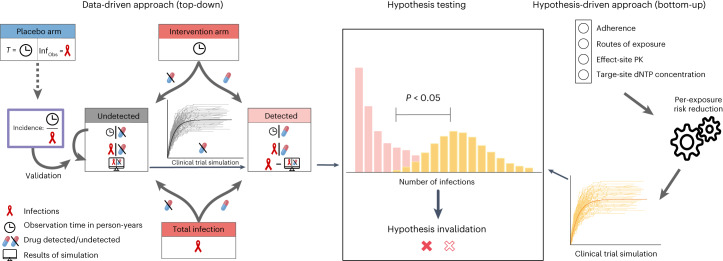
General approach of combining data-driven, ‘top-down’ and hypotheses-driven, ‘bottom-up’ modeling to investigate FTC/TDF-based PrEP efficacy in cis women. In the ‘top-down’ approach, we solely used clinical data to infer PrEP efficacy in cis women with detectable plasma TFV (cis women who took some product). Based on pharmacokinetic models, we could dichotomize PrEP intervention arms. When the drug was undetectable, incidences corresponded to placebo incidences, so efficacy was assumed to be 0%. By simulating this placebo-like subcohort of the PrEP intervention arm, we could estimate drug efficacy in individuals with detectable drug. In the ‘bottom-up’ approach we implemented all previously proposed hypotheses (exposure, drug potency and drug pharmacokinetics) that aim at mechanistically explaining distinct efficacy and adherence–efficacy requirements in cis women (in comparison to MSM) using advanced multiscale modeling and simulation. In a final step, we assessed whether proposed hypotheses hold up against clinically observed outcomes.

In the present study, we used two independent approaches to quantify the adherence–protection relationship for PrEP in cisgender women (Fig. 1). We dichotomized clinical trial data to estimate PrEP efficacy ranges in individuals who took some of the products. We then tested multiple mechanisms that have been proposed to explain adherence–protection relationships in women using an advanced multiscale modeling framework^15–21^. Finally, we evaluated the mechanistic predictions in light of the clinical data, allowing us to statistically rule in or out proposed mechanisms on differential PrEP efficacy in heterosexual women and inform minimal adherence requirements in this risk group. The main findings and policy implications are provided in Table 1.

## Results

### Data-driven, ‘top-down’ analysis of PrEP trials in women

**Fig. 2:**
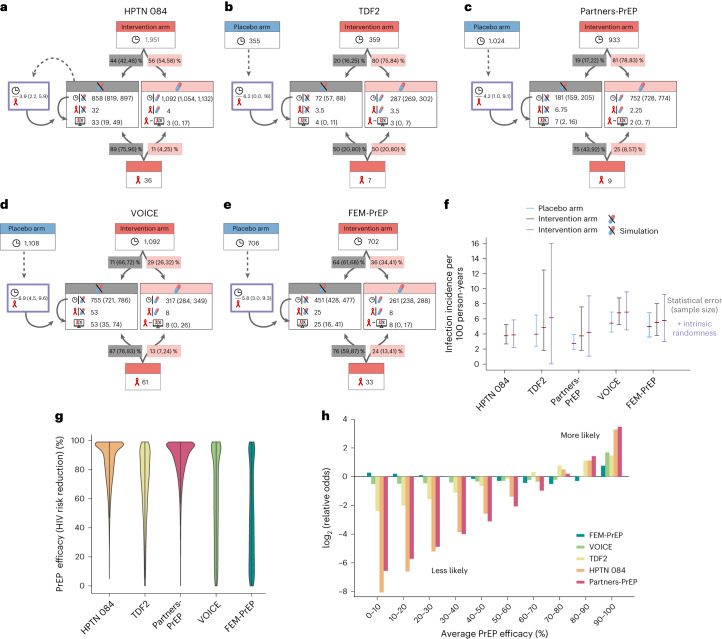
Summary of clinical trials evaluating FTC/TDF-based PrEP in cis women. **a**–**e**, The total observation time in person-years (indicated by clock) in the respective trials (HPTN 084 (**a**), TDF2 (**b**), Parters-PrEP (**c**), VOICE (**d**) and Fem-PrEP (**e**)) dichotomized into ‘detected’ and ‘undetected’ based on the fraction of measurements with detectable plasma TFV. Likewise, the total number of infections (red ribbon) were proportionally assigned based on the fraction of infected individuals who had detectable plasma TFV. We then stochastically simulated the ‘no-drug intervention’ (crossed-out pill, details in Methods) to compute the number of infections in the ‘no-drug intervention’ arm. From both the total observed number of infections and the simulated number of infections in the ‘no-drug intervention’ arm, we could compute the number of infected individuals with detectable plasma TFV. **f**, Incidence in placebo (blue error bars) and ‘drug-undetected’ subcohort of the PrEP intervention arms (gray error bars) with sample sizes indicated in **a**–**e**. Error bars show the mean incidence rates and the 95% confidence intervals (CIs) computed using Wilson’s method. Purple error bars show incidences (and their 95% CI) computed from 10,000 stochastic clinical trial simulations with sampled incidence rate parameters (Methods), depicting uncertainty in the simulation parameter, as well as intrinsic randomness in trial outcomes due to rare events. **g**, Violin plots indicating the probability distributions of average PrEP efficacy from clinical trial simulation only taking data provided in the respective studies (Supplementary Fig. 8). The width indicates the likelihood of a particular efficacy. Square-shaped violin plots indicate uninformative clinical trials (FEM-PrEP, VOICE study), that is, no conclusion can be drawn with regard to the PrEP efficacy, whereas sharply concentrated distributions (HPTN 084 and Partners-PrEP studies) are informative with regard to PrEP efficacy. **h**, Relative odds of particular efficacy ranges (related to **g**).

We simulated five major trials (HPTN 084, FEM-PrEP, VOICE, Partners-PrEP and TDF2 (refs. ^3,11,12,22,23^)) that assessed the impact of FTC/TDF-based PrEP on HIV risk reduction in cisgender women. We first evaluated HIV incidence rates in the respective placebo arms (Fig. 2) and then evaluated the intervention arms. Pharmacokinetic modeling revealed that individuals without detectable plasma tenofovir (TFV) have taken the product at most once weekly (43% probability; interquartile range (IQR): 29–54%) or not at all (Supplementary Figs. 1 and 2). Subsequently, we assumed that individuals with undetectable drug have not taken FTC/TDF at all, hence having 0% PrEP efficacy. The proportion of random samples with undetectable plasma TFV in the FTC/TDF intervention arm was 19% in the Partners-PrEP and TDF2, 44% in HPTN 084, 64% in FEM-PrEP and 71% in VOICE studies (Fig. 2a–e). We assigned this fraction of observation period (person-years) to a ‘drug-undetected’ subcohort, as well as a fraction of the infected individuals, if TFV were undetectable (Fig. 2a–e).

To assess our assumption of 0% efficacy in the ‘drug-undetected’ subcohort, we computed incidences in this subcohort from the respective studies (Fig. 2a–e). These incidences corresponded well with incidences in respective placebo arms, albeit slightly (but not substantially) higher, indicating that we may safely assume 0% efficacy of FTC/TDF in the dichotomized ‘drug-undetected’ subcohort (Fig. 2f). We then simulated the ‘drug-undetected’ subcohort, taking two sources of stochasticity into account: uncertainty in the data-derived incidence rate, as well as intrinsic stochasticity resulting from rare infection events. The resultant number of infections are highly consistent with corresponding clinical data (Fig. 2a–e) and allowed us to quantify uncertainty.

By using the ‘drug-undetected’ simulations, as well as the total number of infections reported in the respective clinical study, we estimated the entire confidence ranges of PrEP efficacy in individuals with detectable drug for the distinct PrEP trials (Methods). These analyses gave three important insights: (1) the VOICE and FEM-PrEP studies span almost the entire theoretically possible range of average PrEP efficacy in individuals taking (some of) the product (see ‘almost uniform distributions’ in Fig. 2g) and are therefore underpowered, because no tendency for any efficacy strata can be deduced (Fig. 2h); (2) the TDF2 study is also relatively uninformative, but rather points toward higher efficacy; and (3) the remaining studies (HPTN 084 and Partners-PrEP) confidently point toward very high PrEP efficacy in women taking some of the product (Fig. 2g), whereby the most likely average PrEP efficacy stratum is 90–100% (Fig. 2h). Importantly, this analysis was solely based on information content of the respective clinical studies and did not yet make any assumption about adherence levels in individuals with detectable plasma TFV.

### Mechanism-based, ‘bottom-up’ modeling

**Fig. 3:**
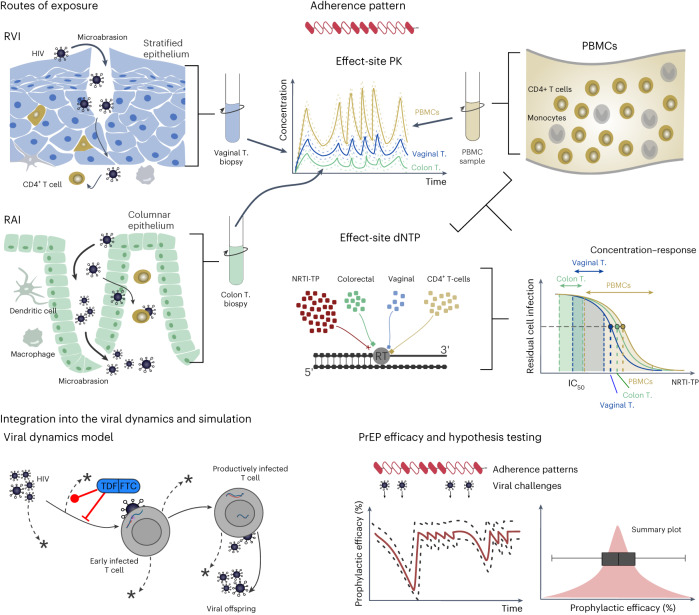
Bottom-up approach for testing the concomitant effects of proposed mechanisms (pharmacokinetics, drug potency and exposure) on PrEP efficacy in heterosexual cis women. Exposure may ocur via either receptive vaginal (RVI) or anal intercourse (RAI). Effect-site drug concentration time-courses were related to either local tissue (T.) biopsies or PBMCs, and via pharmacokinetic models related to adherence patterns in individuals taking PrEP. Concentration–response profiles and drug potency were then computed based on dNTP concentrations in local tissue or CD4^+^ cells and integrated with the effect-site pharmacokinetics to estimate the time-course of drug inhibition of viral replication shortly after exposure. By integrating the viral dynamics with adherence, effect-site pharmacokinetics and drug inhibition, we computed the temporal profile of prophylactic efficacy, that is, the reduction of infection incidence if virus exposure happened at some time *t*. By integrating over all possible times *t*, we derived a summary statistic for an individual with a given adherence and pharmacokinetic profile and an unknown virus exposure time. Final PreP efficacy estimates summarized predictions over many adherence profiles and 1,000 virtual patients (pharmacokinetic parameter sets).

**Fig. 4:**
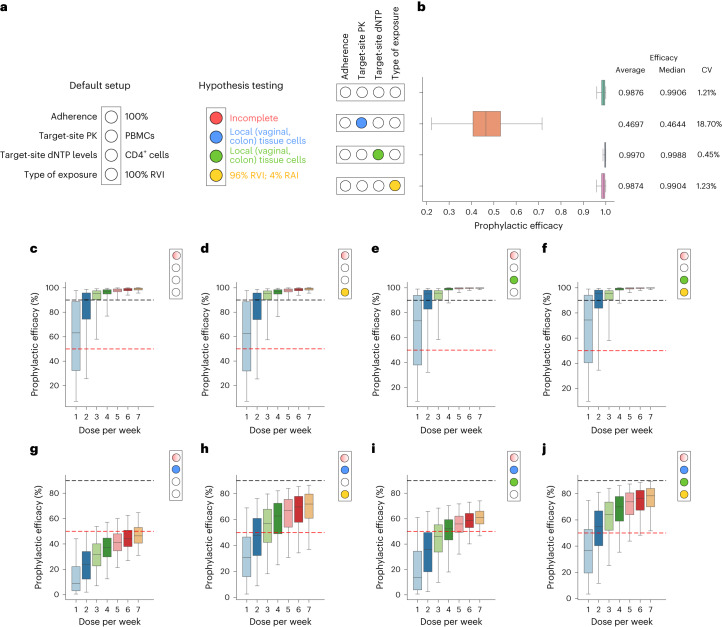
Impact of single and combined mechanisms on PrEP efficacy and adherence requirements of different hypotheses. **a**, ‘Light switch’-based set-up to simulate the impact of hypotheses in isolation or in combination, as described in the main text. **b**, Population prophylactic efficacy estimates considering different hypotheses in isolation (*N* = 1,000 virtual patients were sampled); 200 mg of FTC and 300 mg of TDF were ingested with an adherence level of 100%. The effects of pharmacokinetics at exposure sites, endogenous dNTP level and routes of exposure (receptive anal (RAI) or vaginal intercourse (RVI)) were investigated, respectively. The whisker of the box plot represents 1.5× IQR and CV denotes the coefficient of variation of the average prophylactic efficacy based on 8,400 time points for each of the 1,000 virtual patients. **c**–**j,** Model-predicted prophylactic efficacy if FTC/TDF was taken once, twice, …, 7 d per week on average (**c**). Boxplots show median efficacy and IQR and whiskers extend to 2.5–97.5% range. The 90% and 50% efficacies are highlighted for visual guidance using horizontal dashed black and red lines, respectively. **c**, Baseline scenario with different levels of drug adherence. **d**, Mixed vaginal- and anal exposure. **e**, Altered drug potency at site of exposure (through dNTP levels). **f**, Mixed exposure and altered drug potency. **g**, Drug concentration in local tissue homogenates used as an effect compartment marker. **h**, Concentrations in local tissue homogenates used and mixed exposures occur. **i**, Concentrations in local tissue homogenates and altered drug potency used. **j**, Concentrations in local tissue homogenates, altered drug potency and mixed (anal and vaginal) exposures simulated.

We assessed mechanisms (adherence, exposure-site pharmacokinetics, exposure-site potency and exposure route) that were previously proposed in the context of specific efficacy–adherence profiles in women (Fig. 3). As it is almost impossible to conduct clinical trials that systematically test the influence of these mechanisms on HIV risk reduction, we used integrative (‘bottom-up’) mathematical modeling of available in vitro and ex vivo data^24–30^ to study their potential impact on PrEP efficacy^21^. For simulation, we test proposed mechanisms alone and in combination, in analogy to a light switch (Fig. 4a), where each of the ‘four lights’ can be individually switched on and corresponds to a mechanism that we include in the modeling:

1. Adherence: our Pop-PK (pharmacokinetic) models (Methods) established the link across individual adherence patterns, prodrug (FTC and TFV) concentration–time profiles in the blood plasma, and the pharmacologically active metabolites tenofovir diphosphate (TFV-DP) and emtricitabine triphosphate (FTC-TP) in peripheral blood mononuclear cells (PBMCs). We performed simulations where the adherence is either complete (baseline scenario; light ‘off’) or incomplete (red light ‘on’).
2. Exposure-site pharmacokinetics: we extended our Pop-PK models for putative exposure-site pharmacokinetics, using either FTC-TP or TFV-DP concentrations in PBMCs as a marker for the effect site (blue light ‘off’) or drug concentrations in colorectal and vaginal tissue homogenates (‘blue light on’; Supplementary Fig. 3).
3. Effect-site drug potency: TFV-DP and FTC-TP are both competitive inhibitors of HIV reverse transcription^31^ and hence their potency can be altered by endogenous substrate (deoxynucleoside triphosphate (dNTP)) concentrations^11,12^. We utilized local (vaginal, colorectal) tissue dNTP measurements (‘green light on’) or, in a baseline scenario, used dNTP concentrations in CD4^+^ T cells^32^ (‘green light off’) to estimate exposure-site drug potency and drug combination effects through previously developed models of their molecular mechanisms of action (MMOA)^19,20^.
4. Exposure route: heterosexual cis women may be exposed via receptive anal or vaginal intercourse (RAI or RVI). We either modeled exposure purely via RVI (baseline scenario; ‘yellow light off’) or included 4% anal exposures (‘yellow light on’; Methods).

We simulated TFV and emtricitabine pharmacokinetics^33,34^ following daily intake of FTC/TDF in 1,000 virtual individuals (Supplementary Fig. 4a,b). Intracellular (PBMCs) FTC-TP and TFV-DP concentrations reach their respective steady state after one and seven dosing events, respectively (Supplementary Fig. 4c,d). By mechanistically modeling the intracellular synergistic interaction between TFV and emtricitabine^19^, we calculated the instantaneous efficacy of the drug combination (Supplementary Fig. 5) and based the prophylactic efficacy of FTC/TDF (Fig. 4b) for the baseline scenario on this (individuals are 100% adherent; efficacy markers: PBMC concentrations, dNTP concentrations from CD4^+^ T cells and 100% RVI). This scenario predicted high average prophylactic efficacy (98%) in fully adherent women after RVI. Simulation results for incomplete adherence are depicted in Fig. 4c and show that, if FTC/TDF was taken with an adherence of 14% (once weekly), the median efficacy was 65% (IQR = 35–90%), whereas with two and three doses per week adherence median efficacy climbs to 90% (IQR = 75–96%) and 96% (IQR = 90–98%).

### Impact of individual mechanisms on PrEP efficacy

We next evaluated the individual effects of proposed mechanisms on PrEP efficacy, if individuals fully adhered to the daily FTC/TDF regimen.

When simulating heterosexual exposure with 4% RAI, the overall prophylactic efficacy of once-daily FTC/TDF in fully adherent individuals did not markedly change in comparison to the baseline scenario (only RVI; Fig. 4b).

Based on previously reported^35^ local concentration ratios of FTC-TP to dCTP and TFV-DP to dATP in vaginal, cervical and colonic tissue, we used our previously developed^20^ and validated^36^ MMOA model to compute drug potencies in these tissues (Supplementary Fig. 6). These evaluations indicated that the potency of TFV-DP in the colon would be identical to PBMCs (half-maximal inhibitory concentration (IC_50_) ∼0.1 μM), marginally greater in cervical tissue cells (IC_50_ ∼0.05 μM) and slightly lower in vaginal tissue cells (IC_50_ ∼0.15 μM). The potency of FTC-TP would marginally increase (IC_50_ = 0.39–0.49 μM) in all three tissues, compared with the PBMCs (IC_50_ = 0.85 μM; baseline scenario). Consequently, prophylactic efficacy would be marginally increased (99%) in comparison to the baseline scenario (98%), if local tissue dNTP concentrations were considered (Fig. 4b).

We next investigated the relationship between TFV-DP and FTC-TP concentrations in PBMCs versus local tissue or cell homogenates, and predicted prophylactic efficacy assuming that TFV-DP/FTC-TP concentrations in local tissue homogenates predict the effect. After data extraction and harmonizing (Supplementary Fig. 3), our analysis indicated that TFV-DP concentrations were about 3-fold higher in colon homogenates, compared with PBMCs and 14-fold lower in vaginal tissue. FTC-TP concentrations in colon tissue were 25-fold lower compared with PBMCs and 17-fold lower in vaginal tissue homogenate (Supplementary Fig. 7). Using these data, model-predicted prophylactic efficacy was markedly reduced, that is, the best-case PrEP efficacy in fully adherent individuals was only 47% (IQR = 42–55%), compared with the baseline scenario (efficacy = ∼98%).

In summary, our simulations point out that, if tissue homogenates were a marker for the relevant effect-site concentrations, PrEP efficacy would be markedly reduced, even in fully adherent individuals.

### Combined impact of hypothesized mechanisms on PrEP efficacy and adherence requirements

We simulated all combinations of the aforementioned hypotheses and assessed the impact of incomplete adherence on HIV protection (Fig. 4c–j). In the baseline scenario (Fig. 4c; PBMCs predict effect site), prophylactic efficacy was high (median >95%) when FTC/TDF was taken at least three times a week, on average. Efficacy starts to drop sharply when FTC/TDF was taken once a week, but the median efficacy is still about 65% (IQR = 35–90). Similar results are obtained when mixed RVI and RAI exposures occur, local dNTP levels were altered or these two mechanisms co-occur (Fig. 4d–f). On the contrary, when drug levels in tissue homogenates were considered, drug efficacy dropped considerably, with median efficacy being <50% in fully adherent individuals (compared also in Fig. 4b), dropping gradually to <10% in individuals who take FTC/TDF once a week (Fig. 4g). When both drug and dNTP levels from tissue homogenates were considered (Fig. 4i), the efficacy–adherence profile was elevated by about 10% compared with the aforementioned setting (Fig. 4g). On consideration of mixed RVI and RAI exposures, drug concentrations in homogenates with and without altered dNTP concentrations yielded similar efficacy–adherence profiles (Fig. 4h,j); median achievable prophylactic efficacy in fully adherent women would be <80% and gradually decreases ∼50% if FTC/TDF were taken 2 d a week on average and ∼30% when taken FTC/TDF once a week.

In summary, if drug concentrations in local homogenates were the relevant marker for prophylactic efficacy, then FTC/TDF would incompletely protect women from HIV infection, even in fully adherent individuals. In all scenarios where local tissue concentrations were considered, incomplete adherence has a gradual effect on prophylactic efficacy. In contrast, if drug concentrations in PBMCs were the relevant concentration marker for PrEP, HIV protection would be high, as long as individuals took PrEP ≥3× a week (Fig. 4c–f).

### Challenging mechanism-based modeling with clinical data

To rule out proposed hypotheses corroborating risk-group-specific adherence requirements in women, we tested whether the ‘bottom-up’-inferred average PrEP efficacies (Fig. 4b–j) resulted in infection numbers that are inconsistent with clinical data (Fig. 1). The TDF2, FEM-PrEP and VOICE studies do not allow us to distinguish any of the hypotheses (Table 2) as already suggested by Fig. 2g,h. By comparing our simulations with the remaining clinical trials (Partners-PrEP and HPTN 084; Table 2), we observed that any simulation scenario in which drug concentrations in PBMCs were used as a marker of efficacy (Fig. 4c–f) was generally in agreement with reported clinical outcomes. On the contrary, if local (vaginal, colorectal) drug concentrations were considered as a marker for prophylactic efficacy, corresponding clinical trial simulations were either statistically incongruent with clinical data (*P* < 0.05) or statistically unlikely (*P* < 0.1), (Table 2 and Fig. 4g–j). This finding strongly argues that drug concentrations in PBMCs, and not local tissue concentrations, are a more appropriate marker for determining PrEP efficacy in cisgender women.

**Table 1:** Policy box

**Background**	Globally, most HIV infections occur in heterosexual cis women in resource-limited settings. In this risk group, self-protection with generic FTC/TDF-PrEP could constitute a major pillar for HIV prevention. However, clinical trials in cis women had seemingly inconsistent outcomes, sparking uncertainty about adherence requirements and reluctance in testing on-demand PrEP regimen. Although MSM may take on-demand PrEP, it is currently recommended that women take FTC/TDF daily, which may negatively impact on PrEP uptake and adherence behavior in cisgender women
**Main findings and limitations**	We reanalyzed all FTC/TDF-PrEP trials and found that outcome variability can solely be explained by the proportion of trial participants not taking the prescribed drugs. Moreover, we found that PrEP efficacy is consistently high (∼90%) in individuals taking some of the product. In a ‘bottom-up’ approach, we modeled hypotheses corroborating risk-group-specific adherence–efficacy profiles and challenged proposed hypotheses with the clinical data. We found that hypotheses of putative cisgender women/MSM differences either do not impact on efficacy or significantly underpredicted clinical efficacy. The most consistent models suggested that adherence–efficacy profiles in women are similar to those in MSM The present study is limited to assessing PrEP adherence requirements in women, although additional aspects induced by risk-group-specific PrEP uptake and adherence (pill-taking) behavior are probably relevant to the success of PrEP in women
**Policy implications**	Our multiscale modeling approach provides strong evidence of high PrEP efficacy in cis women taking some product, whereas mechanistic hypotheses proposing more restrictive PrEP adherence requirements in women compared with MSM are not supported by clinical trial data or by the suggested mechanisms. To advance HIV prevention in women, more actionable FTC/TDF regimens in women should be identified and obstacles to PrEP adherence investigated

Overall, our simulations highlight that VOICE, FEM-PrEP and TDF2 studies are underpowered to evaluate average PrEP efficacy, because either the overall observation time was too short (TDF2: 359 person-years) or too few individuals took the study drugs (29% in FEM-PrEP and 36% in VOICE). Both factors contribute to too little observation time in individuals taking some of the product (Fig. 2b,d,e). Consequently, reasoning on specific adherence requirements in cisgender women based on these studies may be statistically unsupported. When the remaining studies are dichotomized for ‘drug undetected’ (≤1 dose per week) versus ‘drug detected’ (≥1 dose per week), PrEP efficacy estimates are ∼90% in women (Fig. 2g–h). Notably, any model involving local drug pharmacokinetics in vaginal and colon tissue homogenates substantially underpredicted this level of efficacy, even when adherence was complete (Fig. 4). From our simulations, the most consistent scenario is the one where intracellular drug concentrations (TFV-DP and FTC-TP) in PBMCs predict effect-site concentrations (in exposure-site resident CD4^+^ cells) and thus oral prophylactic efficacy.

## Discussion

Through comprehensive analysis of available clinical data, combined with computational modeling of FTC/TDF-based PrEP: (1) we assessed whether apparent discrepancy among clinical trial outcomes^3,9,11,12,23,37^ in women has a statistical foundation; (2) we challenged various mechanisms that were proposed in the context of risk-group-specific efficacy; and (3) we analyzed adherence requirements that provide sufficient PrEP protection.

Our population-pharmacokinetic (Pop-PK) modeling indicated that individuals with undetectable TFV levels (clinical adherence marker) must have taken FTC/TDF less than once a week, if at all (Supplementary Figs. 1 and 2). As TFV detectability has been reported in a random subset of the PrEP intervention arm of PrEP trials in cisgender women, we were able to dichotomize the intervention arms into subcohorts ‘undetectable drug’ (≤1 dose per week) and ‘detectable drug’ (≥1 dose per week). It is interesting that identical dichotomization of MSM trials concluded that FTC/TDF-based PrEP is ∼92% efficient in individuals who take some product, in contrast to un-dichotomized estimates of 44% in MSM^38^.

When we assumed negligible PrEP efficiency for the ‘undetectable-drug’ subcohort and simulated the corresponding trials, we derived incidences closely matching incidences in the respective placebo arms. Although serving as an internal control for our analysis, this finding indicated that the intervention arm of the distinct studies contains variable ‘placebo-like’ observation periods, being individuals who were either never protected or not protected for a period of time. This analysis alone could explain the bulk of apparent discrepancy between clinical studies with FTC/TDF-based PrEP in women. In essence, studies that reported low average PrEP efficacies (VOICE and FEM-PrEP studies) also contained little or no information about the actual intervention, because most participants did not take the drugs (64% in FEM-PrEP and 71% in VOICE studies, based on dichotomization). Corresponding observation times in individuals taking some product translate into 261 (FEM-PrEP study) to 317 (VOICE study) person-years, making these studies underpowered for assessing the effect strength of the actual PrEP intervention. This analysis raises the question of whether individuals not taking the product should be identified in a timely manner with adherence markers, excluded from the intervention arm and be replaced by new study recruits. On the other hand, Partners-PrEP and HPTN 084 studies entailed considerable observation time in individuals taking some of the product (752 and 1,092 person-years, respectively), which consistently translates into high PrEP efficacy.

But, how frequently does FTC/TDF need to be taken by cisgender women? Previously, various mechanisms have been proposed to determine the adherence–efficacy profiles in women that allow us to challenge them with the clinical evidence. More specifically, we tested hypotheses related to exposure-site pharmacokinetics, exposure-site potency and exposure route. Our observation was that, if PBMCs were the relevant matrix measuring effect-site drug concentrations, PrEP efficacy was high (>90%) if FTC/TDF was taken three to four times a week. Notably, in these simulations the FTC component would substantially contribute to FTC/TDF’s effect, whereas we conservatively estimate TDF’s contribution. When local tissue drug concentrations were considered as the relevant matrix, the maximum achievable efficacy in fully adherent individuals was 50–80%, depending on the combination of tested hypotheses. This drop in efficacy was mainly due to ∼16-fold depletion of TFV-DP and FTC-TP in vaginal tissue compared with PBMCs (Supplementary Fig. 7). As these predictions were inconsistent with clinical data (Table 2), we found the mechanistic explanation that often referred to women having intrinsically different adherence requirements because of local drug levels^35^ unlikely. We concluded that either tissue pharmacokinetic data lack predictive power with regard to the effect site or our current understanding of exposure-site pharmacokinetics is insufficient. On the other hand, our modeling suggested that PBMCs may be a suitable surrogate marker (Table 2). Notably, both matrices (PBMCs, tissue homogenate) have advantages and disadvantages. Local tissue biopsies contain a homogenate of many cell types not relevant to HIV infection, as well as low amounts of tissue-resident CD4^+^ cells. As cellular uptake and intracellular activation into FTC-TP/TFV-DP depend on the cell-specific expression of involved membrane transporters and intracellular kinases^39,40^, drug concentrations in tissue homogenates reflect an average over a very heterogeneous mix (‘cocktail’) that may poorly correlate with concentrations in tissue-resident CD4^+^ cells. On the other hand, PBMCs contain a large proportion of relevant HIV target cells (CD4^+^ T cells)^41^ and have been shown to correlate with drug concentrations in CD4^+^ cell populations^42–45^. However, they are a systemic marker and, during oral PrEP, FTC/TFV reaches the effect site (exposure-site-resident CD4^+^ cells) through the systemic circulation. Hence, PBMC-contained CD4^+^ cells, as well as exposure-site-resident CD4^+^ cells, may encounter similar extracellular FTC/TFV concentrations that can be taken up and converted into active intracellular moieties. In contrast, PBMCs may not be a suitable effect-site marker for topically applied drugs, which enter tissue-resident CD4^+^ cells from the putative exposure site with negligible systemic drug levels.

In contrast to the modeling study in ref. ^35^, we found a modest impact of dNTP-to-nucleoside reverse transcriptase inhibitors (NRTIs)-TP (substrate-to-inhibitor) concentration ratios in tissue on FTC/TDF potency (Methods*)*. One reason is that the drug effect equation used in previous work by others^35^ scales directly with the substrate-to-inhibitor ratio. Notably, the direct effects of TFV-DP and FTC-TP have been well studied^46^ and reproducible kinetic parameters deduced (summarized in ref. ^18^), all of which indicate that the direct scaling used in this previous work^35^ may be incorrect owing to inhibitor-binding saturation.

A recent publication implied higher adherence requirements in women compared with MSM based on analysis of the HPTN 084 versus HPTN 083 studies^47^, although there was actually no statistical difference in any adherence category. Notably, utilized adherence strata were based on TFV-DP levels in dried blood spots (DBSs) derived from a cohort of individuals who regularly took a proportion of the drugs (for example, every third day). This adherence pattern may, however, be quite different from the adherence patterns in clinical trials. In addition, because TFV-DP in DBS has a fourfold longer half-life (about 17 d) compared with PBMCs^48,49^, the drug may still be detectable in DBSs, when there are no longer protective levels in PBMCs. In combination with decreasing adherence behavior over the duration of the present study, which was frequently observed in women^50–52^ and infrequent drug testing, it is thus possible that the recent analysis overcalled some of the three infections in women into moderate adherence categories (2–3, 4–6 doses per week). Although HPTN 084 clearly shows that none of the individuals who acquired HIV had perfect adherence (Supplementary Table 1), the aforementioned arguments may indicate that the adherence–risk reduction profile could in fact be steeper than suggested by ref. ^47^. Only 4 of 36 infected individuals in HPTN 084 showed some evidence of product taking around the time of infection (TFV-DP in DBSs ≥ 350 fmol per punch, corresponding to ∼1 dose per week^49^; Supplementary Table 2), whereas most seemed to have taken the product before a clinical visit, but not otherwise (detectable plasma TFV levels and DBS TFV-DP <350 fmol per punch), suggesting poor adherence behavior in women in the present study^53^.

Another recent study^54^ analyzed data from VOICE and Partners-PrEP studies based on risk strata and plasma TFV. They suggested that ‘low-risk’ MSM and women have identical adherence requirements, whereas ‘high-risk’ women require higher adherence than MSM. Although the risk categories have been criticized^55,56^, the VOICE dataset exclusively contributes to the group of cisgender women. This dataset is characterized by higher baseline incidence than the Partners-PrEP study (Fig. 2f) and, moreover, poor adherence in the ‘drug-detected’ subgroup^54^. Consequently, high-risk, infected women have low drug concentrations, possibly coinciding with high baseline incidence contributed by the VOICE dataset. This may have confounded the comparison in ref. ^54^.

In summary, our investigation of FTC/TDF-based PrEP efficacy in women highlights that apparent discrepancy between clinical trials can largely be attributed to different proportions of non-PrEP-covered periods within the respective intervention arms. When dichotomizing the clinical trials accordingly, we found that mechanistic models utilizing pharmacokinetics in PBMCs predict oral prophylactic efficacy, without regard for colon–vaginal differences. If this was also the case for MSM, then adherence requirements between women and MSM^42,57^ may not be different (Supplementary Fig. 13) and observed MSM versus women differences in PrEP effectiveness could rather be related to specific adherence (pill-taking) behavior in cis women in these studies^50–52^. A rational way forward would hence identify obstacles to PrEP uptake and properly address them to unfold the full potential of FTC/TDF-based PrEP in cisgender women.

However, our model-based estimates require confirmation by demonstrating concurrence of the model predictions in MSM studies, as well as formal clinical assessment of an on-demand regimen in cisgender women. Finally, our work contains a number of limitations: the dichotomization into ‘drug undetected’ versus ‘drug detected’ averages over all adherence levels in the ‘drug-detected’ subcohort. Unlike other authors^42,47,57^, we choose this crude categorization to increase statistical power when estimating average PrEP efficacies. Yet, still, all studies except the Partners-PrEP and HPTN 084 ones were underpowered after dichotomization. Notably, introduction of further adherence strata for the Partners-PrEP and HPTN 084 studies would make their analysis underpowered, too^47^. Our dichotomy-derived average PrEP efficacy would be uninformative with regard to critical adherence levels if most individuals in the ‘drug-detected’ group were highly adherent (for example, 6–7 pills a week). Although dosing frequency in the Partner-PrEP study may have been high^58^, Anderson et al.^47^ suggest that most HPTN 084 participants took rather few pills a week. As both studies translate into high average PrEP efficacy (Fig. 2g,h), the results from the HPTN 084 study suggest some adherence insensitivity in women, in these studies. Unlike others^54^ we did not analyze risk factors other than the route of exposure. Also, although infectivity after colorectal challenge may be higher in cisgender women than in MSM^59^, further studies may be warranted to increase confidence in our interpretations. Last, since protection from infection decreases with increasing HIV exposures^18^, what we term ‘PrEP efficacy’ in bottom-up analysis is strictly defined as the per-exposure risk reduction.

## Methods

### Pharmacokinetics of FTC/TDF

We use pharmacokinetic models to assess adherence benchmarks in clinical studies, as well as in mechanistic modeling of PrEP efficacy.

The two components of FTC/TDF are prodrugs. FTC is taken up by cells and triphosphorylated intracellularly, where it acts as an analog of deoxycytosine triphosphate (dCTP). TDF is first metabolized to TFV by first-pass liver metabolism and then twice phosphorylated in cells to form TFV-DP, a deoxyadenosine triphosphate (dATP) analog.

To fully reflect the pharmacokinetics of the two drugs, we utilized the previously developed models by Burns et al.^33^ and Garrett et al.^34^. In both models, the amount of (pro-)drug in the dosing compartment (*D*), the amount of circulating compound in the central (blood plasma, *A*_1_) and the peripheral (*A*_2_) compartments, as well as the amount of pharmacologically active metabolite (TFV-DP and FTC-TP, respectively) in the cellular compartment (*A*_3_; PBMCs) is considered. We calculated in units of micromoles internally to avoid unit conversions. The following ordinary differential equations (ODEs) were used to describe the mass flux between aforementioned compartments, in between two dosing events:

1 $$\frac{{\mathrm{d}}}{{\mathrm{d}}t}D=-{k}_{a}\times D\qquad({\rm{dosing}})$$

2 $$\begin{array}{l}\displaystyle\frac{{\mathrm{d}}}{{\mathrm{d}}t}{A}_{1}={k}_{a}\times D-{k}_{12}\times {A}_{1}+{k}_{21}\times {A}_{2}-{k}_{e}\times {A}_{1}\\\qquad\quad\ -{f}_{13}({A}_{1})+{f}_{31}({A}_{3})\qquad\qquad\quad\ ({\rm{dosing}})\end{array}$$

3 $$\frac{{\mathrm{d}}}{{\mathrm{d}}t}{A}_{2}={k}_{12}\times {A}_{1}-{k}_{21}\times {A}_{2}\qquad({\rm{peripheral}})$$

4 $$\frac{{\mathrm{d}}}{{\mathrm{d}}t}{A}_{3}={f}_{13}({A}_{1})-{f}_{31}({A}_{3})-{f}_{30}({A}_{3})\qquad({\rm{cell}})$$

The terms *k*_a_ and *k*_e_ (1/h) denote the absorption and elimination rate constants, respectively. The terms *k*_12_ and *k*_21_ (1/h) are the influx and outflux rate constants to/from the peripheral compartment, respectively. For the emtricitabine model, we used ${f}_{13}=\frac{{V}_{\max }\times {A}_{1}}{{K_{\mathrm{m}}}+{A}_{1}}$, *f*_30_ = 0 and *f*_31_ = *k*_31_ × *A*_3_, where *V*_max_ (μmol h^−1^) and *K*_m_ (μmol) denote the parameters for the nonlinear cellular uptake and intracellular conversion of FTC to FTC-TP and *k*_31_ denotes the rate constant of intracellular dephosphorylation of FTC-TP to FTC and its efflux into the circulation.

For the TFV model, we used *f*_13_ *=* *k*_13_ × *A*_1_, *f*_30_ = *k*_30_ × *A*_3_ and *f*_31_ = 0, where *k*_13_ and *k*_31_ denote the rate constants of uptake, phosphorylation versus efflux and dephosphorylation, respectively.

Between dosing events, the system of ODEs was numerically integrated using scipy.integrate.solveivp in Python. At a dosing event, *τ*_dose_, the amount of the drug in the dosing compartment *D* was elevated using the dosed amount (μmol). Concentrations of the respective plasma prodrug concentrations and intracellular metabolite concentrations were derived by dividing by the respective volumes, that is, ${C}_{1}={{\mathrm{A}}}_{1}/{V}_{1}$ and ${C}_{3}={A}_{3}/{V}_{3}=I$.

Pharmacokinetic parameter values for 1,000 virtual patients were sampled from the distributions described in refs. ^33,34^ and are given in Supplementary Data Files 1 and 2. In line with the literature, we assumed that the two drugs do not interact with regard to their pharmacokinetics.

Adherence profiles were simulated by randomly drawing dosing events with a probability that corresponds to the weekly average dosing frequency.

### Estimation of incidence rate from clinical data

We assume that the number of observed infections during a clinical trial is binomially distributed, so the probability that *n* infections occurred in a clinical trial with *N* total participants can be calculated as:

5 $$P({N}_{{\mathrm{Inf}}}=n)=\left(\begin{array}{c}N\\ n\end{array}\right)\cdot {({P}_{{\mathrm{Inf}}})}^{n}\cdot{(1-{P}_{{\mathrm{Inf}}})}^{N-n}$$

where ${P}_{{{\mathrm{Inf}}}}\in \left[\mathrm{0,1}\right]$ denotes the probability that a single individual becomes infected during the course of the clinical study. ${P}_{{{\mathrm{Inf}}}}$ can be represented by the parameters given in the respective clinical studies as ${P}_{{{\mathrm{Inf}}}}=\frac{n}{N}=\frac{{T}_{{{\mathrm{total}}}}}{N}\times {r}_{{{\mathrm{Inf}}}}$, where ${T}_{{{\mathrm{total}}}}$ represents the observation time (typically in person-years) and ${r}_{{{\mathrm{Inf}}}}\in \left[0,\frac{N}{{T}_{{{\mathrm{total}}}}}\right]$ denotes the incidence rate (1/person-years), the distribution of which we want to express analytically. After plugging ${P}_{{{\mathrm{Inf}}}}$ into $P({N}_{{{\mathrm{Inf}}}}=n)$ and normalizing, we can get the cumulative density function (CDF):

6 $$F({r}_{{\mathrm{Inf}}})=\displaystyle\frac{{\displaystyle\int }_{0}^{{r}_{{\mathrm{Inf}}}}{x}^{n}\times {(N-{T}_{{\mathrm{total}}}\times x)}^{N-n}{\mathrm{d}}x}{{\displaystyle\int }_{0}^{\frac{N}{{T}_{{\mathrm{total}}}}}{x}^{n}\times {(N-{T}_{{\mathrm{total}}}\times x)}^{N-n}{\mathrm{d}}x}$$

The number of observed infections *n*, as well as the number of participants, *N*, and the observation period *T*_total_ were reported for each clinical study. Therefore, the infection incidence *r*_Inf_ can be sampled using the inverse transform sampling with the CDF^60^ derived above. The infection incidence for each clinical study is shown in Fig. 2f (Results).

### Clinical trial simulation (basic model)

We simulated the different clinical trials evaluating PrEP efficacy in cis women using Monte-Carlo simulations (Gillespie simulations). We set up a simple stochastic model with two reactions that simulate infection and drop out in the respective trials:

7 $${R}_{1,{\mathrm{trial}}}:S\mathop{\longrightarrow }\limits^{{r}_{{\mathrm{inf}}}}I\qquad{R}_{2,{\mathrm{trial}}}:S\mathop{\longrightarrow}\limits^{{r}_{d{\mathrm{r}}-{\mathrm{out}}}}\varnothing$$

where *S* (‘susceptibles’) is initialized with the number of individuals in the respective clinical trial arm, the rate *r*_inf_ is set either to the incidence rate in the placebo arm (for simulation of the placebo arm or ‘drug-undetected’ subcohort), or to the incidence in the placebo arm, multiplied by 1 − φ (PrEP efficacy) to simulate interventions. The drop-out rate ${r}_{{{\mathrm{d}}r}-{{\mathrm{out}}}}$ is reciprocally related to the follow-up time in the respective clinical trial arm, that is, ${{\mathrm{Average}}\; {\mathrm{follow-up}}\; {\mathrm{time}}\; {\mathrm{per}}\; {\mathrm{person}}}=\frac{1}{{r}_{{{\mathrm{d}}r}-{{\mathrm{out}}}}+{r}_{\inf }}$. The average follow-up time per person is calculated from the number of individuals and the total observation time in person-years. Derived parameters are depicted in Fig. 2a–e (main text).

### Top-down estimation of PrEP efficacy

For each clinical trial, we dichotomized the intervention arm into two groups: individuals who probably did not take the drug (‘drug undetected’ in Fig. 2; main text) and individuals who took some of the product (‘drug detected’). We then simulated the ‘drug-undetected’ subcohort with corresponding incidence and drop-out rates (calculated from the number of observed infections, the population size and the total follow-up in this subcohort; Fig. 2f). Incidences in the placebo arms and the ‘drug-undetected’ subcohort of the respective intervention arms were identical (Fig. 2f). Thus, we could safely assume 0% PrEP efficacy in individuals without detectable plasma TFV.

From simulations we derived estimates for the number of infected individuals in the ‘drug-undetected’ subcohort ‘Inf_sim_ (drug undetected)’. The number of infected individuals with detectable drug levels was then calculated as:

8 $${\mathrm{Inf}}_{{\mathrm{Clin}}\_{\mathrm{sim}}} ({\mathrm{drug}}\; {\mathrm{detected}}) = {\mathrm{Inf}}_{\mathrm{Total}} - {\mathrm{Inf}}_{\mathrm{sim}} ({\mathrm{drug}}\ {\mathrm{undetected}})$$

with values shown in Fig. 2a–e. By running many stochastic simulations, we finally derived the probability distribution of the number of infected individuals in the ‘drug detected’ subcohort *P*_Inf_, as illustrated in Supplementary Fig. 8a. Using this approach, we thus did not have to make any assumption about PrEP efficacy to derive estimates of the number of infected individuals in the subcohort with detectable plasma TFV.

Next, we wanted to estimate the PrEP efficacy in individuals with detectable drug from the clinical studies. To achieve this goal, we simulated the subcohort of individuals with detectable drug. We took the corresponding number of participants and observation time. Then, a PrEP efficacy level was randomly sampled from a uniform distribution, ${\varphi }{i \sim }{\mathscr{U}}(0,1)$, and the incidences were appropriately scaled ${r}_{{{\mathrm{Inf}}}}\left({\varphi }_{i}\right)={r}_{{{\mathrm{Inf}}}}\times (1-{\varphi }_{i})$. We then ran 1,000,000 stochastic simulations, with *φ*_*i*_ sampled from ${\mathscr{U}}(0,1)$ in each simulation. We collected the resultant number of infections ${{{\mathrm{In}}}{{\mathrm{f}}}_{{{\mathrm{sim}}}}}(\varphi _{i})$ and computed the distribution of efficacy for each generated infection number $P\left(\varphi _{i}|{{\mathrm{Inf}}}\right)$, as depicted in Supplementary Fig. 8b.

Finally, we combined the results from the two steps to derive the probability distribution over the PrEP efficacy that explains the clinical data (Supplementary Fig. 8c). Mathematically, the distribution of efficacy is calculated as:

9 $$P\left({\varphi }_{i}\right)=\sum _{{{\mathrm{Inf}}}}P_{{\mathrm{Inf}}}\times P\left({\varphi }_{i},|,{{\mathrm{Inf}}}\right)$$

The estimated probability distributions of PrEP efficacy for each clinical study are shown in Fig. 2g,h and the main text.

### Pharmacokinetics in exposed tissue

We identified seven publications that report local TFV-DP or FTC-TP concentrations in cisgender women and cisgender men^24–30^, using different dosing regimens and measurement time points, contributing to eighteen datasets in total. To enable the comparison between the datasets, we simulated the corresponding dosing schedules using the pharmacokinetic models exemplified above (Supplementary Fig. 3a,b). We used this step as an internal control of our pharmacokinetic models, with regard to pharmacokinetic profiles in PBMCs, as well as to check for consistency between the different studies.

For each study and dosing regimen, where both local and PBMC drug levels were reported, we computed the fold deviation between measured mean (or median) drug concentrations ${\hat{C}}_{{{\mathrm{PBMC}}},{{\mathrm{obs}}}}({t}_{{{\mathrm{obs}}}})$ in PBMCs and the corresponding prediction from simulating our Pop-PK models ${\hat{C}}_{{{\mathrm{PBMC}}},{{\mathrm{sim}}}}({t}_{{{\mathrm{obs}}}})$.

10 $${\rm{Fold}}\,{\rm{deviation}}=\frac{{\hat{C}}_{{\mathrm{PBMC}},{\mathrm{obs}}}({t}_{{\mathrm{obs}}})-{\hat{C}}_{{\mathrm{PBMC}},{\mathrm{sim}}}({t}_{{\mathrm{obs}}})}{{\hat{C}}_{{\mathrm{PBMC}},{\mathrm{sim}}}({t}_{{\mathrm{obs}}})}$$

This analysis revealed remarkable consistency between the studies and between our model and reported concentration measurements in PBMCs. For TFV-DP, concentration measurements in nine of ten datasets fell within a twofold deviation of our corresponding model predictions. One single-dose regimen from Thurman et al.^30^ showed a greater than twofold deviation from the corresponding simulations (Supplementary Fig. 9a). For FTC-TP, seven of eight datasets fell within a twofold deviation. One once-weekly regimen from the HPTN066 study yielded an ∼15-fold different concentration compared with our corresponding simulation (Supplementary Fig. 9b). As these 2 (out of 18) datasets deviated substantially from our model and from the other 16 studies, they were excluded during the downstream inference of local FTC-TP and TFV-DP levels.

We then computed local-to-PBMC concentrations at the study-specific measurement time points. Computing these ratios assumes that the kinetics (that is, half-life) of the drugs in the tissues and the PBMCs are similar. However, as there was no systematic trend with regard to local site-to-PBMC concentration ratios across different dosing regimens (single dose, multiple dose, shortly and long after last dosing), the assumption of proportional kinetics in PBMCs and local tissues seemed appropriate. The local site-to-PBMC drug concentration ratios were calculated as a weighted geometric mean (Supplementary Fig. 3c).

11 $$\begin{array}{l}{\mathrm{log}}({\mathrm{Weighted}}\,{\mathrm{geometric}}\,{\mathrm{mean}}\,{\mathrm{local-to-PBMC}}\,{\mathrm{ratio}})\\=\displaystyle\frac{{\sum }_{i=1}^{S}\sqrt{{N}_{i}}\times {\mathrm{log}}_{2}({r}_{i})}{{\sum }_{i=1}^{S}\sqrt{{N}_{i}}}\end{array}$$

where ${r}_{i}=\frac{{\hat{C}}_{{{\mathrm{PBMC}}},{{\mathrm{obs}}}}\left({t}_{{{\mathrm{obs}}}}\right)}{{\hat{C}}_{{{\mathrm{PBMC}}},{{\mathrm{sim}}}}\left({t}_{{{\mathrm{obs}}}}\right)}$ denotes the ratio between measured local concentrations and simulated concentrations in the PBMCs with the corresponding dosing regimen and at the corresponding observation time *t*_obs_. The weight $\sqrt{{N}_{i}}$ considers the statistical error inherent to each study, where ${N}_{i}$ is the sample size for study *i*, which was set to ${N}_{i}=1$, if the number was not stated in the respective publication. Below the limit of quantification (BLQ), data were excluded in our analysis and reported median values were considered only if at least 50% of the measurements were above the lower limit of quantification (LLOQ). Obtained local site-to-PBMC concentration ratios are depicted in Supplementary Fig. 7 for the different matrices: colorectal tissue panels A and B, cervical tissue in panels C and D and vaginal tissue in panels E and F. Overall, concentration ratios agree well between different studies and across dosing regimens. In particular, the fact that concentration ratios are consistent across different dosing regimens supports the assumption that pharmacokinetics (for example, half-life) are proportional between PBMCs and the distinct local sites. As cervical tissue and vaginal tissue concentration ratios were similar, but vaginal tissue concentrations were more abundant and less variable (Supplementary Fig. 7), we subsequently used the colon-to-PBMC and the vaginal-to-PBMC concentration ratios in simulations. The final concentration ratio estimates (Supplementary Table 3) were then used as conversion factors, that is, multiplied with the concentrations in PBMCs (Supplementary Fig. 3e).

### Pharmacodynamics of FTC/TDF

The intracellular triphosphorylated moieties FTC-TP and TFV-DP are NRTIs that compete with endogenous nucleotides (dCTP and dATP, respectively) for incorporation into nascent proviral DNA during the reverse transcription of the viral RNA genome. Once incorporated, reverse transcription is (temporarily) halted, because FTC-TP and TFV-DP lack the necessary chemical group to attach the next incoming nucleotide during reverse transcription. In a previous work^19^, we evaluated the combinatorial effect of FTC-TP and TFV-DP by extending a model for the molecular mechanism of action (MMOA) of NRTIs for various drug–drug interaction hypotheses. The refined model acknowledges the fact that the combination therapy appears to decrease dNTP pools in vivo^61^, which would favor NRTI incorporation and results in synergistic inhibition^19^, whereas other mechanisms of interaction were found to be negligible at clinically relevant drug concentrations.

In this work, to speed up computation, we precalculated the combinatorial effects $\eta \left({I}_{1},{I}_{2}\right)$ using the MMOA model for a 100 × 100 log-spaced grid of drug concentrations ranging from 0.001 μM to 150 μM for each of the two nucleoside reverse transcriptase inhibitors ${I}_{1}$, ${I}_{2}$. During PK–pharmacodynamic (PD) simulations we then derived $\eta \left({I}_{1}\left(t\right),{I}_{2}\left(t\right)\right)$ for any combination of drug concentrations ${I}_{1}\left(t\right)={C}_{3,{{\mathrm{TFV}}}-{{\mathrm{DP}}}}\left(t\right)$ and ${I}_{2}\left(t\right)={C}_{3,{{\mathrm{FTC}}}-{{\mathrm{TP}}}}\left(t\right)$ encountered at time $t$ by interpolating on the precalculated grid (scipy.interpolate.griddata^62^ in Python).

### Drug potency in exposed tissue

As outlined in the main text, a putative hypothesis for male/female differences in FTC/TDF-based PrEP is that the drugs may have different potency (for example, IC_50_) in the vaginal and colorectal tissues. As both TFV-DP and FTC-TP are competitive inhibitors, putative differences in endogenous dNTPs at exposure sites may alter their potency. To assess the influence of dNTP levels in colorectal vaginal and cervical tissues on FTC-TP and TFV-DP potency, we utilized the local tissue dNTP concentrations reported in Cottrell et al.^35^ (Fig. 1 therein), in the MMOA model^36^. Resultant concentration–response curves and changes in drug potency are shown in Supplementary Fig. 6.

### HIV replication model and PK–PD link

To estimate infection and infection prevention by PrEP, we used a previously developed model of HIV replication^15,17,63^. In brief, the model consists of free viruses (Vs), as well as early and late infected T cells (${T}_{1}$ and ${T}_{2}$, respectively). The replication cycle is modeled by six reactions with propensities ${a}_{1-6}$ (equations (12)–(17)) that model the following processes: Vs can be cleared by the immune system or by unsuccessful infection of T cells (reaction ${R}_{1}$). This process is altered by NRTIs (like TFV-DP and FTV-TP)^18,36,64,65^ in the sense that the drugs increase the probability of unsuccessful infection. Preintegrated virus in early infected T cells (${T}_{1}$) can be cleared (reaction ${R}_{2}$) and late infected T cells (${T}_{2}$) can be cleared in reaction ${R}_{3}$. Vs can also successfully infect T cells to form early infected T cells with reaction ${R}_{4}$. This reaction, too, is altered by the presence of NRTIs^18,36,64,65^, that is, the drugs inhibit the process of cell infection by inhibiting reverse transcription of the virus genome^18,36^. In case of successful infection (and reverse transcription), proviral DNA may be integrated into the host DNA to form a late infected T cell (reaction ${R}_{5}$), which produces new viral progeny with reaction ${R}_{6}$. Utilized parameters are found in ref. ^21^ (Table 2 therein). For HIV replication, we have the following reaction stoichiometries and reaction propensities (* refers to an elimination reaction):

12 $${R}_{1}:V\to \ast \quad{a}_{1}({I}_{1},{I}_{2})=\left({\mathrm{CL}}+\left[\frac{1}{{\rho }_{{\mathrm{rev}},\varnothing }}-(1-\eta ({I}_{1},{I}_{2}))\right]\times \beta \times {T}_{u}\right)\times V$$

13 $${R}_{2}:{T}_{1}\to \ast \qquad{a}_{2}=({\delta }_{{\mathrm{PIC}}}+{\delta }_{{T}_{1}})\times {T}_{1}$$

14 $${R}_{3}:{T}_{2}\to \ast \qquad{a}_{3}={\delta }_{{T}_{2}}\times {T}_{2}$$

15 $${R}_{4}:V\to {T}_{1}\qquad{a}_{4}({I}_{1},{I}_{2})=(1-\eta ({I}_{1},{I}_{2}))\times \beta \times {T}_{u}\times V$$

16 $${R}_{5}:{T}_{1}\to {T}_{2}\qquad{a}_{5}={k}\times {T}_{1}$$

17 $${R}_{6}:{T}_{2}\to V+{T}_{2}\qquad{a}_{6}={N}_{T}\times {T}_{2}$$

**Table 2:** Number of infected individuals for different hypotheses and comparison with clinical trial data

Scenario									
PrEP efficacy (bottom-up)	91.1 (29.3, 99.9)	91.0 (28.9, 99.9)	93.4 (34.8, 100)	93.5 (37.0, 100)	35.8 (2.6, 60.4)	58.0 (12.4, 84.3)	48.2 (3.3, 71.5)	65.0 (15.7, 87.6)	Study
Inf_clin_sim_	**3 (0, 17)**								**HPTN 084**
Inf_sim_	4 (1, 9)	4 (1, 9)	3 (0, 7)	3 (0, 7)	27 (15, 42)	18 (9, 29)	22 (12, 35)	15 (7, 25)	
*P* value	0.5	0.4996	0.5007	0.4911	0.0204	0.0903	0.0483	0.1399	
Inf_clin_sim_	**3 (0, 7)**								**TDF2**
Inf_sim_	1 (0, 5)	1 (0, 6)	1 (0, 4)	1 (0, 4)	11 (2, 27)	7 (1, 18)	9 (1, 22)	6 (1, 16)	
*P* value	0.3986	0.3975	0.3487	0.3460	0.1412	0.2586	0.1936	0.3246	
Inf_clin_sim_	**2 (0, 7)**								**Partners-PrEP**
Inf_sim_	3 (0, 8)	3 (0, 8)	2 (0, 6)	2 (0, 6)	20 (7, 41)	14 (4, 28)	16 (5, 34)	11 (3, 24)	
*P* value	0.4412	0.4437	0.5106	0.5116	0.0204	0.0606	0.0357	0.0972	
Inf_clin_sim_	**8 (0, 26)**								**VOICE**
Inf_sim_	2 (0, 5)	2 (0, 6)	1 (0, 4)	1 (0, 4)	14 (7, 23)	9 (4, 17)	12 (5, 20)	8 (3, 15)	
*P* value	0.3102	0.3123	0.2952	0.2881	0.2958	0.4448	0.3766	0.5039	
PrEP efficacy (bottom-up)	93.1 (38.8, 99.9)	93.0 (38.4, 99.9)	95.1 (45.4, 100)	95.2 (47.4, 100)	37.6 (3.9, 60.9)	60.0 (16.1, 84.6)	50.4 (5.1, 71.8)	67.1 (20.5, 87.8)	**FEM-PrEP**
Inf_clin_sim_	**8 (0, 17)**								
Inf_sim_	1 (0, 4)	1 (0, 4)	1 (0, 3)	1 (0, 3)	9 (3, 17)	6 (1, 12)	7 (2, 14)	5 (1, 10)	
*P* value	0.2171	0.2193	0.2029	0.2039	0.4529	0.4435	0.5122	0.3976	

The first row depicts the distinct hypotheses in ‘traffic-light’ notation and the second row the ‘bottom-up’ estimated mean PrEP efficacy (95% CI) in individuals with detectable plasma TFV for that hypothesis (compare Fig. 4). The columns show the mean number of infected individuals (95% CI), from ‘top-down’ clinical trial simulation, Inf_clin_sim_ (depicted in bold; compare Fig. 1), and from ‘bottom-up’ simulation, Inf_sim_, with deduced PrEP efficacies for the distinct hypotheses. The *P* value tests for differences in the number of infected individuals deduced from bottom-up modeling versus clinical data. The *P* value was empirically calculated by computing the proportion of 10^6^ simulation pairs, for which the null hypothesis was true (that is, *H*_0_: *P* = no. of simulations where infected individuals from hypothesis X was equal to or less than clinical estimate/total no. of simulations; *H*_1_: no. of infected individuals from hypothesis X more than the corresponding clinical estimate). Crosses visually indicate whether the statistical test provided trends (single unfilled red cross) or statistically different predictions at *P* < 0.05 (filled single- or double-red cross).

### Infection prevention (prophylactic efficacy)

Having modeled the time-dependent effects of FTC/TDF on viral replication, we can estimate prophylactic efficacy using a recently developed numerical method in a matter of seconds^21^.

The prophylactic efficacy, $\varphi$, is herein defined as the relative reduction in infection probability for a prophylactic regimen $S$, compared with the infection probability in the absence of prophylaxis, ⌀, after virus challenge ${Y}_{t}$:

18 $$\varphi ({Y}_{t},S)=1-\frac{{P}_{I}({Y}_{t},S)}{{P}_{I}({Y}_{t},\varnothing )}$$

where ${P}_{I}\left({Y}_{t},S\right)$ and *P*_I_(*Y*_*t*_,⌀) denote the infection probabilities in the presence and absence of a prophylactic regimen $S$, if a given viral exposure, ${Y}_{t}$, occurs at time $t$. In the present study, a prophylactic regimen refers to a pharmacokinetic profile that is a consequence of a history of drug dosing, as well as individual pharmacokinetic parameters (Supplementary Data Files 1 and 2). For the absence of prophylaxis, *P*_I_(*Y*_*t*_,⌀), analytical solutions have been presented in ref. ^63^. To estimate the probability of infection (and prophylactic efficacy), it is mathematically more convenient to compute the extinction probability, ${P}_{{\mathrm{E}}}$, which is its complement:

19 $${P}_{{\mathrm{I}}}\left({Y}_{t},S\right)=1-{P}_{{\mathrm{E}}}\left({Y}_{t},S\right)$$

To compute the extinction probability for a certain regimen, we used the method developed in ref. ^21^:

20 $$\begin{array}{cc}\frac{{\mathrm{d}}{P}_{{\mathrm{E}}}\left({Y}_{t}=\widehat{V}\right)}{{{\mathrm{d}}t}} & ={a}_{1}\left(t\right)\times \left[{P}_{{\mathrm{E}}}\left({Y}_{t}=\widehat{V}\right)-1\right]\\ & +{a}_{4}\left(t\right)\times \left[{P}_{{\mathrm{E}}}\left({Y}_{t}=\widehat{V}\right)-{P}_{{\mathrm{E}}}\left({Y}_{t}=\widehat{{T}_{1}}\right)\right]\\ \frac{{\mathrm{d}}{P}_{{\mathrm{E}}}\left({Y}_{t}=\widehat{{T}_{1}}\right)}{{{\mathrm{d}}t}} & ={a}_{2}\times \left[{P}_{{\mathrm{E}}}\left({Y}_{t}=\widehat{{T}_{1}}\right)-1\right]\\ & +{a}_{5}\times \left[{P}_{{\mathrm{E}}}\left({Y}_{t}=\widehat{{T}_{1}}\right)-{P}_{{\mathrm{E}}}\left({Y}_{t}=\widehat{{T}_{2}}\right)\right]\\ \frac{{\mathrm{d}}{P}_{{\mathrm{E}}}\left({Y}_{t}=\widehat{{T}_{2}}\right)}{{{\mathrm{d}}t}} & ={a}_{3}\times \left[{P}_{{\mathrm{E}}}\left({Y}_{t}=\widehat{{T}_{2}}\right)-1\right]+{a}_{6}\times \left[{P}_{{\mathrm{E}}}\right.\left({Y}_{t}=\widehat{{T}_{2}}\right)\\ & -{P}_{{\mathrm{E}}}\left({Y}_{t}=\widehat{{T}_{2}}\right)\times {P}_{{\mathrm{E}}}\left.\left({Y}_{t}=\widehat{V}\right)\right]\end{array}$$

where the time-dependent reaction rates ${a}_{1}\left(t\right)$ and ${a}_{4}\left(t\right)$ are computed according to equations (12) and (15). The system of ordinary differential equation (20) is solved backwards using standard ODE solvers as outlined in ref. ^21^.

### Modeling heterosexual virus exposure

We investigated two modes of heterosexual exposures: receptive anal (RAI) vs vaginal intercourse (RVI). In simulations that (1) consider local tissue drug and dNTP concentrations, differences between RAI and RVI arise due to differences in drug concentration, as well as drug potency at the two sites. Moreover, (2) the amount of virus being transmitted and translocated to a physical site that enables productive viral replication (the inoculum size ${Y}_{t0}$) is different for the two types of exposures. In simulations, we consider higher inoculum sizes for RAI, because the physiological barrier that separates donor virus from acceptor target cell environments only constitutes a single layer of epithelial cells (compare Fig. 3).

We have previously developed exposure models for RAI^18^. In this work, the number of transmitted viruses that translocate to a physical site that enables productive viral replication (the inoculum size, ${Y}_{t0}$) was drawn from a binomial distribution ${Y}_{t0}$ ∼ В(VL, *r*_RAI_), where we drew the virus load in the donor VL from a log(normal distribution) (details in ref. ^18^, Supplementary Text S1 therein) and derived the ‘success rates’ *r*_RAI_, such that average infection rates for unprotected sexual intercourse (${\widehat{P}}_{{\mathrm{I}}}$) coincide with reported values^7,66^ (Supplementary Figs. 10 and 11). For a purely receptive vaginal intercourse, we parametrized *r*_RVI_ accordingly using average infection rates for unprotected vaginal exposure^60,66–71^. The generated inoculum size distribution used in our models is depicted in Supplementary Fig. 10b. The corresponding parameters of our exposure model are: *r*_RAI_ = 3.7 × 10^−3^ and *r*_RVI_ = 9.1 × 10^−5^ (Supplementary Fig. 10a) and log(VL) ∼ *N*(µ, σ) with µ = 4.51 and σ = 0.98 (ref. ^18^). These exposure models allowed us to reproduce realistic infection probabilities for unprotected intercourse and incorporate them into the bottom-up modeling to estimate the efficacy of PrEP.

Moreover, we also investigated the ratio of RAI among total sexual acts in heterosexual cis women. As the estimation of this ratio varies in different studies^72–74^, we investigated different RAI frequencies π_RAI_ in the range 1–5%. It has been previously reported that 40% of heterosexual transmissions in cis women can be attributed to RAI^32,72^. The following equation allows then to estimate the (average) frequency of RAI:

21 $$0.4=\frac{{\pi }_{{\mathrm{RAI}}}\times {\widehat{P}}_{{\mathrm{I}}}({\mathrm{RAI}},\varnothing )}{{\pi }_{{\mathrm{RAI}}}\times {\widehat{P}}_{{\mathrm{I}}}({\mathrm{RAI}},\varnothing )+(1-{\pi }_{{\mathrm{RAI}}})\times {\widehat{P}}_{{\mathrm{I}}}({\mathrm{RVI}},\varnothing )}$$

where $\widehat{{P}_{{\mathrm{I}}}}\left({{\mathrm{RAI}}},\varnothing \right)$ and $\widehat{{P}_{{\mathrm{I}}}}\left({{\mathrm{RVI}}},\varnothing \right)$ denote the average infection probability for each of unprotected anal and vaginal sexual intercourse, respectively (Supplementary Fig. 11). With these values, we can calculate the equation above and obtain the value of ${\pi }_{{{\mathrm{RAI}}}}$ ranging from 1% to 3.6%. Based on these results, we will take an upper boundary of 4%, that is, in simulations that consider anal and vaginal receptive intercourse to be 4% of exposures simulated as RAI.

### Clinical trial simulation using bottom-up inferred PrEP efficacy

Clinical trial simulation was done as explained before using parameters from the respective clinical trial ‘drug-detected’ subcohorts (Fig. 2a–e). In these simulations incidence rates were scaled by the mechanistically inferred (adherence-) averaged PrEP efficacy, $\widehat{\varphi }$, from the distinct hypotheses ${r}_{{{\mathrm{Inf}}}}\left(\widehat{\varphi }\right)={r}_{{{\mathrm{Inf}}}}\times (1-\widehat{\varphi })$ (see also Fig. 4c–j). The (adherence-)averaged PrEP efficacy was calculated as:

22 $$\widehat{\varphi }=\mathop{\sum }\limits_{j=1}^{7}{\widehat{\varphi }}_{j}\times \frac{{p}_{j}({\mathrm{TFV}} > {\mathrm{LLOQ}})}{{\sum }_{j=1}^{7}{p}_{j}({\mathrm{TFV}} > {\mathrm{LLOQ}})}$$

where *j* denotes the number of pills that were taken per week (on average), ${\widehat{\varphi }}_{j}$ denotes average efficacy value for this adherence level and ${p}_{j}({{\mathrm{TFV}}} > {{\mathrm{LLOQ}}})$ is the probability that the TFV plasma concentration for adherence level *j* is above the LLOQ, as depicted in Supplementary Figs. 1–2. The distribution of $\frac{{p}_{j}({{\mathrm{TFV}}} > {{\mathrm{LLOQ}}})}{{\sum }_{j=1}^{7}{p}_{j}({{\mathrm{TFV}}} > {{\mathrm{LLOQ}}})}$ for each *j* is depicted in Supplementary Fig. 12. The number of infections derived in this fashion were statistically compared with the ones derived from the dichotomized clinical data, as depicted in Table 2 (main text).

### Statistical analysis

PrEP efficacy estimates in Table 2 were statistically compared based on empirically computed *P* values to test for differences in the number of infected individuals deduced from bottom-up modeling versus clinical data. The *P* value was empirically calculated by computing the proportion of 10^6^ simulation pairs, for which the null hypothesis was true, for example, P(*H*_0_) = no. of simulations where infected individuals from hypothesis X were less than or equal to clinical estimate/total number of simulations; P(*H*_1_) = no. of infected individuals from hypothesis X were greater than corresponding clinical estimate/total number of simulations. Standard statistical tests were not used, because, given the sample size of 10^6^ simulations, they would lead to large type I errors.

### Infection events in HPTN 084

We analyzed data from the 36 infected individuals in the FTC/TDF arm of the HPTN 084 trial^3^, which are provided in ref. ^31^ (Supplementary Appendix therein). For each infected individual E1–E36, concentration measurements of tenofovir in the blood plasma, as well as TFV-DP concentrations in DBSs around the time of infection, are provided (Supplementary Table 1 for individuals with some evidence of drug intake). In the present study, the limit of detection was 0.31 mg ml^−1^ of plasma tenofir (≈0.001 μM), as well as 31.3 fmol per punch for DBS samples. The reference adherence–concentration benchmarks are reported in Supplementary Table 2.

### Reporting summary

Further information on research design is available in the Nature Portfolio Reporting Summary linked to this article.

## Online content

Any methods, additional references, Nature Portfolio reporting summaries, source data, extended data, supplementary information, acknowledgements, peer review information; details of author contributions and competing interests; and statements of data and code availability are available at 10.1038/s41591-023-02615-x.

### Supplementary information

Supplementary Information Supplementary Figs. 1–13 and Tables 1–3.

Reporting Summary

Supplementary Data 1 Supplementary Dataset 1 Emtricitabine pharmacokinetic parameters. Supplementary Dataset 2 Tenofovir pharmacokinetic parameters.

## Data Availability

Individual pharmacokinetics data are included in the Supplementary Data. All other datasets used in the analysis are included in the published code at https://github.com/KleistLab/PrEP_TruvadaWomen with 10.5281/zenodo.8370715 (ref. ^75^).

## Code availability

All programs have been implemented in Python 3 using the SciFy and the matplotlib package and are freely available at https://github.com/KleistLab/PrEP_TruvadaWomen with 10.5281/zenodo.8370715 (ref. ^75^).
