# Supplementary material for: Model-based predictions of protective HIV pre-exposure prophylaxis adherence levels in cisgender women
Source: Nat Med. 2023 Nov 13;29(11):2753–62. doi: 10.1038/s41591-023-02615-x (PMC10667095; doi:10.1038/s41591-023-02615-x)
Supplement: Supplementary file 1 — Supplementary Figs. 1–13 and Tables 1–3. [file 41591_2023_2615_MOESM1_ESM.pdf]

nature medicine

Article

https://doi.org/10.1038/s41591-023-02615-x

Model-based predictions of protective HIV pre-exposure prophylaxis adherence levels in cisgender women

In the format provided by the authors and unedited

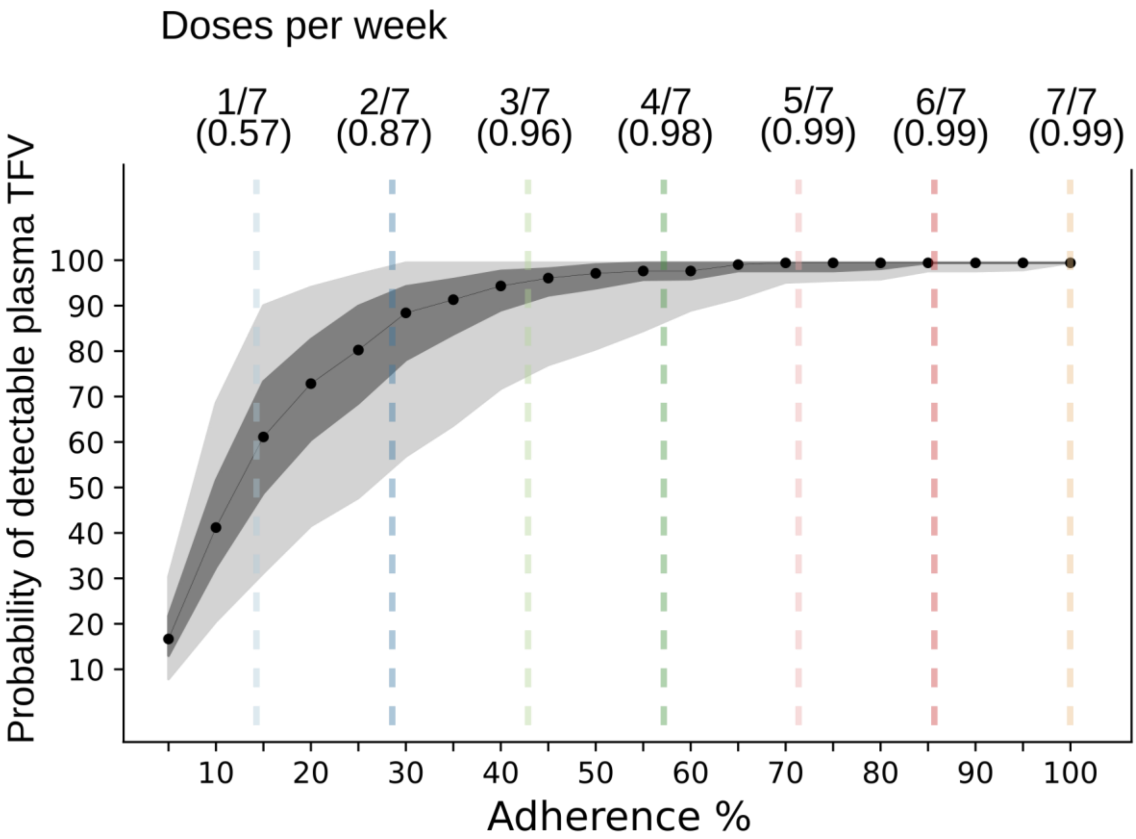

Supplementary Figure S1: Probability of detectable plasma TFV (LLOQ = 0.001µM) for different adherence levels. For each *average* adherence level, dosing profiles were randomly sampled. Plasma tenofovir (TFV) pharmacokinetis for oral 300mg TDF dosing were then simulated in 1000 virtual patients using our pharmacokinetic models (*Methods* section). For each adherence level, the percentage of time points where the plasma TFV was above the lower limit of quantification (LLOQ = 0.001µM) were then calculated. The solid lines indicate the median, whereas the dark- and light grey areas present the quartile range and the 2.5% − 97.5% range respectively. The vertical dashed lines indicate the adherence level with one-, two-, …, seven doses per week *on average*. The numbers above the plot represent the number of doses per week and the probability that plasma TFV is detectable. LLOQ = 0.001µM is related to clinical studies HPTN 084, Partners-PrEP, TDF2 and VOICE[3,12,22,23] .

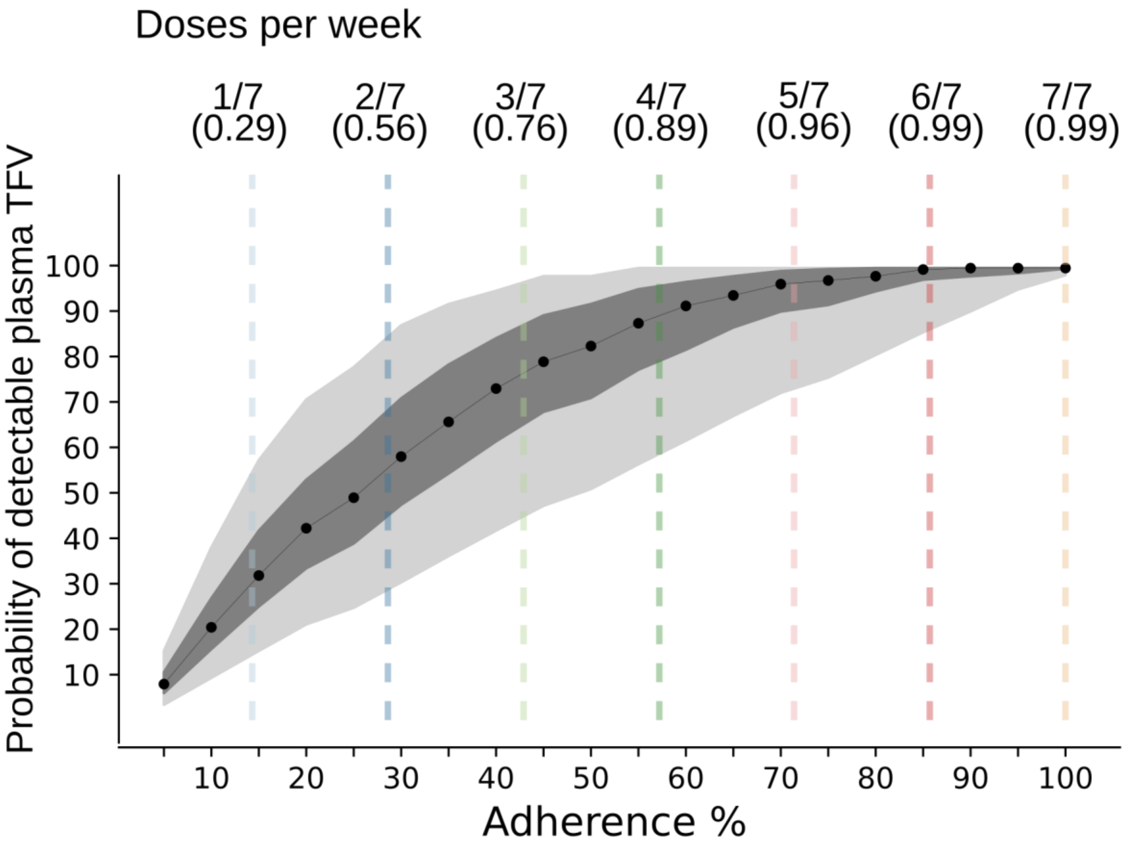

Supplementary Figure S2: Probability of detectable plasma TFV (LLOQ = 0.035µM) for different adherence levels. For each *average* adherence level, dosing profiles were randomly sampled. Plasma tenofovir (TFV) pharmacokinetis for oral 300mg TDF dosing were then simulated in 1000 virtual patients using our pharmacokinetic models (*Methods* section). For each adherence level, the percentage of time points where the plasma TFV was above the lower limit of quantification (LLOQ = 0.035µM) were then calculated. The solid lines indicate the median, whereas the dark- and light grey areas present the quartile range and the 2.5% − 97.5% range respectively. The vertical dashed lines indicate the adherence level with one-, two-, …, seven doses per week *on average*. The numbers above the plot represent the number of doses per week and the probability that plasma TFV is detectable. LLOQ = 0.035µM is related to the FEM-PrEP study[11].

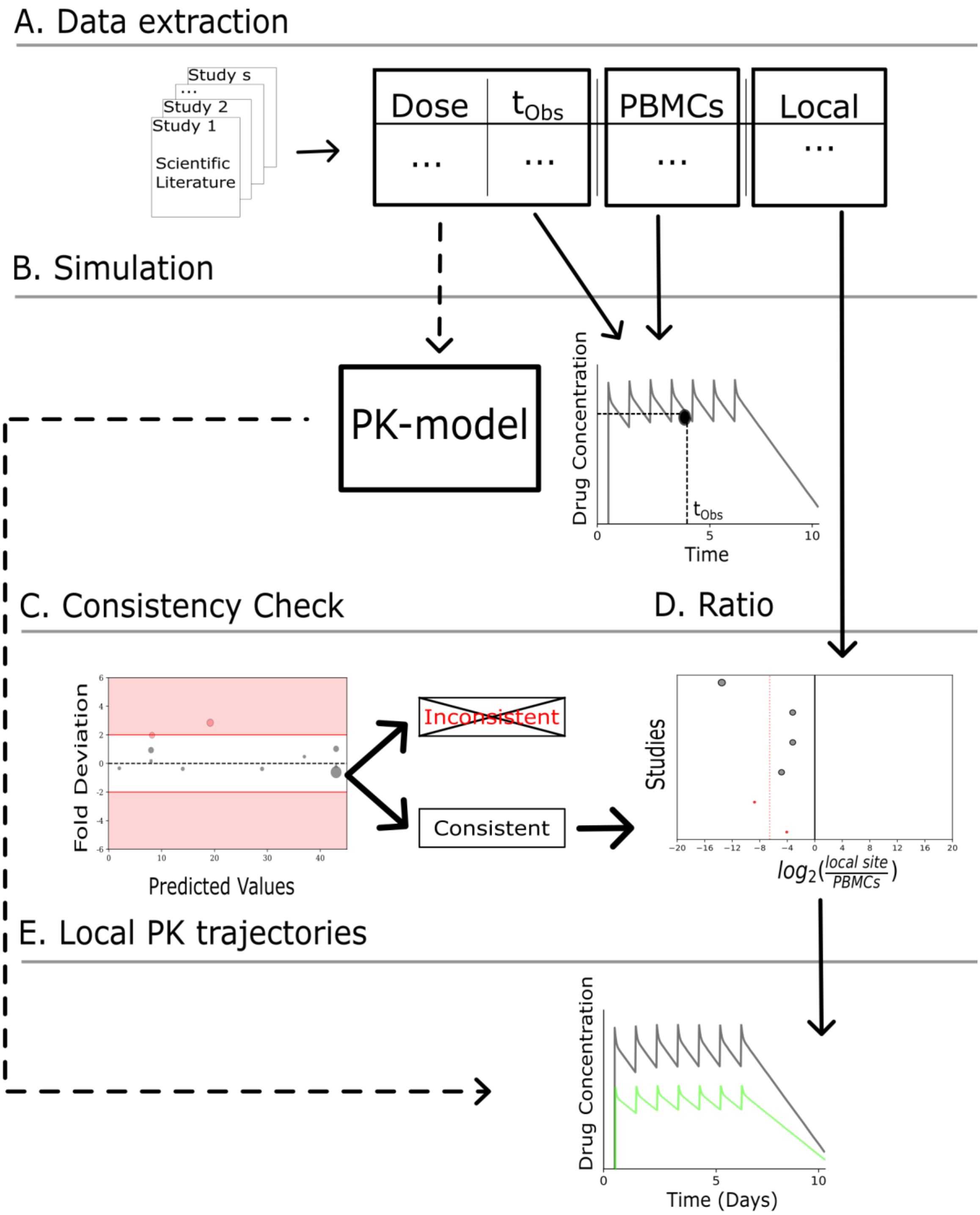

Supplementary Figure S3: Workflow for the derivation of local concentration time profiles. (A) Data was extracted from the literature. (B) Study-specific dosing regimens and measurement time points were simulated to derive corresponding PBMC concentrations. This step allowed to compare data from different studies. (C) A consistency check is carried out between the observed and the predicted PBMCs concentrations. When consistent with other studies and with our simulations, the studies were included in the subsequent steps. (D) Weighted geometric means of the local-to-PBMC concentration ratios across available studies were computed. (E) The local-to-PBMC concentration ratios were used to infer drug pharmacokinetics in local tissues (green) from pharmacokinetics in PBMCs (black).

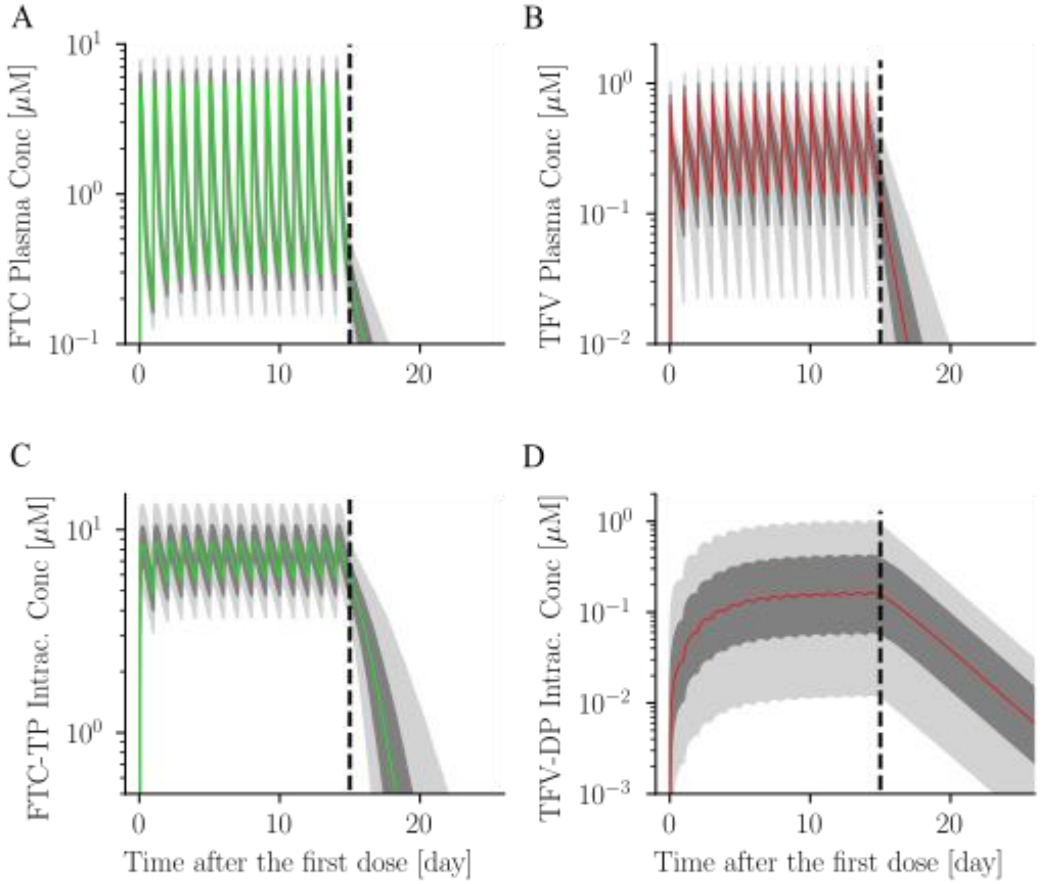

Supplementary Figure S4: Population pharmacokinetic simulations of oral daily FTC/TDF. Population pharmacokinetic simulations for seven days oral 200/300mg dosing every 24hours in 1000 virtual patients. A & B: concentrations of the circulating prodrug (FTC, TFV), C & D: concentration of the intracellular active moiety (FTC-TP, TFV-DP) in PBMCs. The solid lines indicate the median, whereas the dark- and light grey areas present the quartile range and the 2.5% − 97.5% range respectively. The vertical dashed lines indicate the discontinuation of drug dosing after seven days.

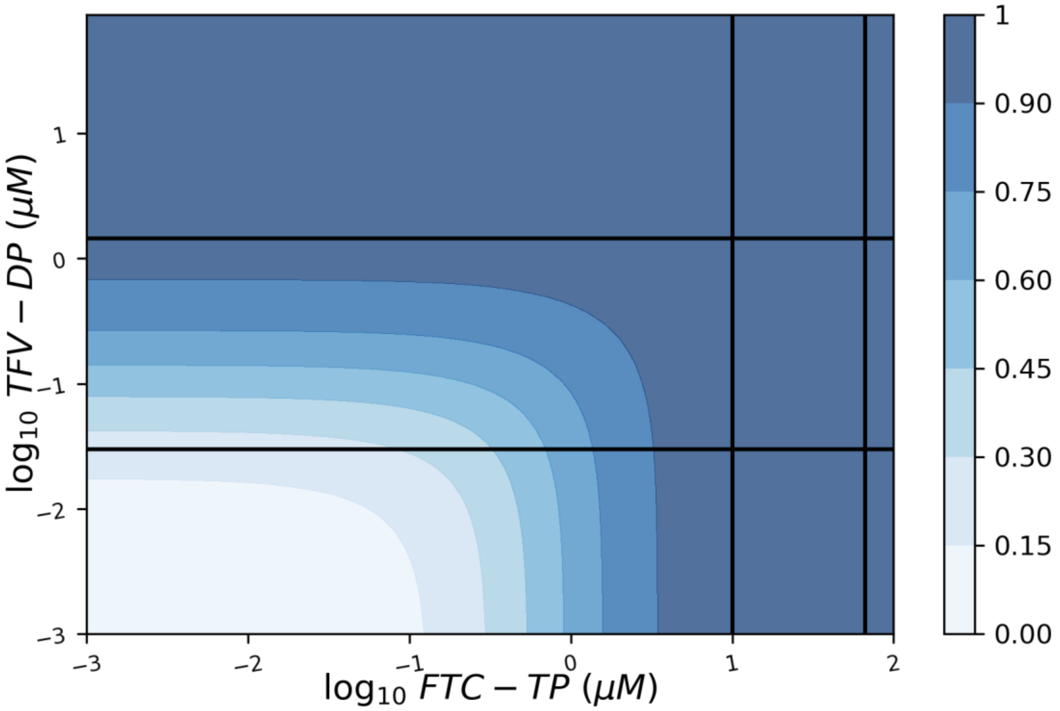

Supplementary Figure S5: Combined effect of FTC-TP and TFV-DP. The combination effect (colour bar) at clinically relevant concentrations of FTC-TP (concentration range: 0.03-1.44μM) and TFV-DP (concentration range: 10-66.66μM) was computed from a molecular mechanisms of action model (MMOA, *Methods* section). In the MMOA model, FTC-TP and TFV-DP molecularly interact by depleting the concentrations of endogenous deoxynucleotide levels, leading to a synergistic inhibition of reverse transcription. Computed values from the MMOA model have been interpolated to allow rapid calculation of their combined effects during PrEP simulations.

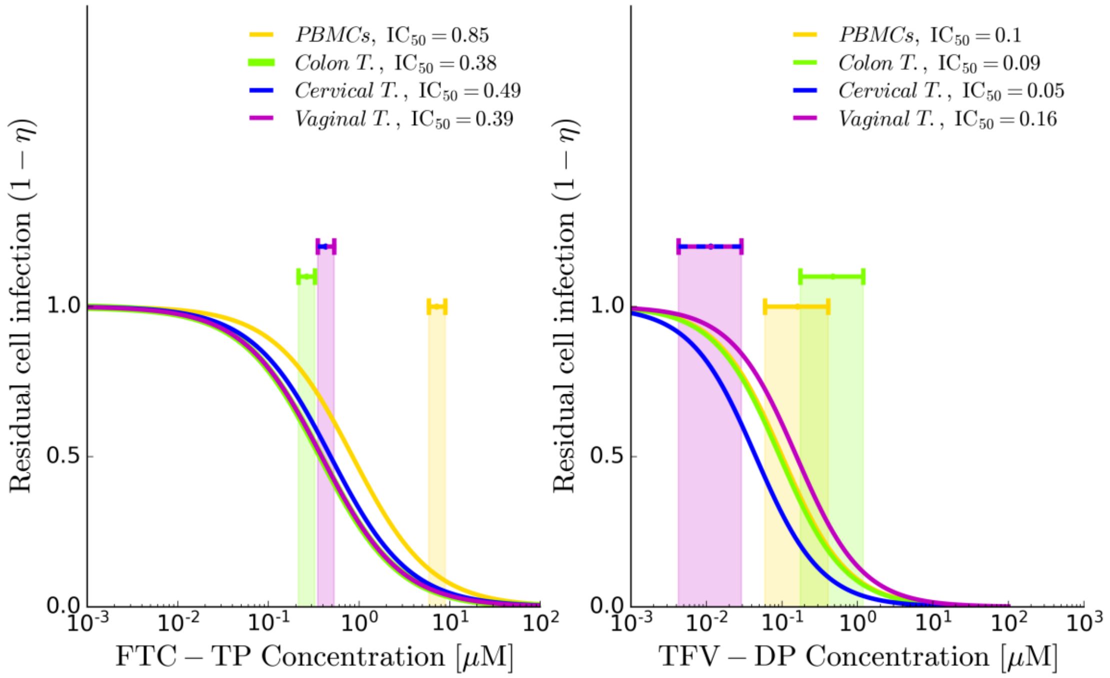

Supplementary Figure S6: MMOA-predicted concentration-response curves for FTC-TP. (A) and TFV-DP (B). The reference MMOA output is shown in yellow, where the dNTP concentration refer to intracellular concentrations in resting CD4+ T-cells (Table 1 in[19]). For the tissue-specific concentration-response curves, tissue- specific dNTP concentrations were used as reported in Cottrell et al.[35]: Colon Tissue, dATP = 0.052, dCTP = 0.028 µM (green line); Cervical Tissue: dATP = 0.238, dCTP = 0.342 µM (blue line); Vaginal Tissue, dATP = 0.273, dCTP = 0.09 µM (magenta line). The horizontal error bars represent simulated median drug concentrations and interquartile ranges in local tissues at steady state in n = 1000 virtual individuals who are fully adherent to one daily oral FTC/TDF (200/300mg).

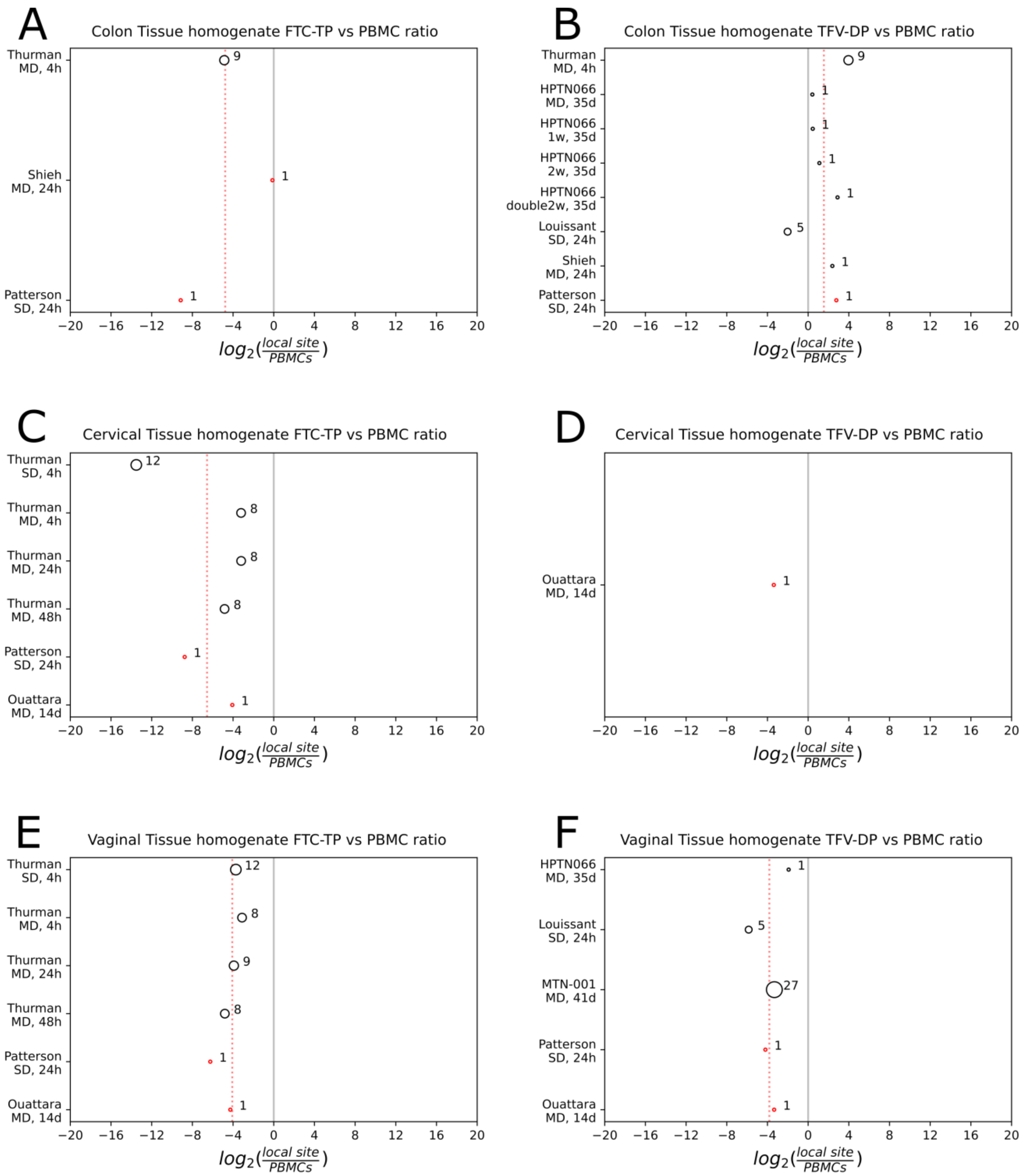

Supplementary Figure S7: Tissue homogenate-to-PBMC conentration ratios. A-B: Concentration ratios in colon tissue homogenates for FTC-TP (A) and TFV-DP (B); C-D: Concentration ratios in cervical tissue homogenates for FTC-TP (C) and TFV-DP (D); E-F: Concentration ratios in vaginal tissue homogenates for FTC-TP (E) and TFV-DP (F). The dotted red line denotes the weighted geometric mean and the grey line indicates a ratio equal to one (equal levels of drug at the local site and in the PBMCs). For each study, the dosing scheme is reported (multiple doses (MD) or single dose (SD), one, two or four tablets per week (1w, 2w, 2*2w)) along with the sampling time. The data points in red indicated studies for which concentrations in PBMCs were not validated with our models, because they were not reported in the original work. The markers are scaled proportionally to the sampling error (√N).

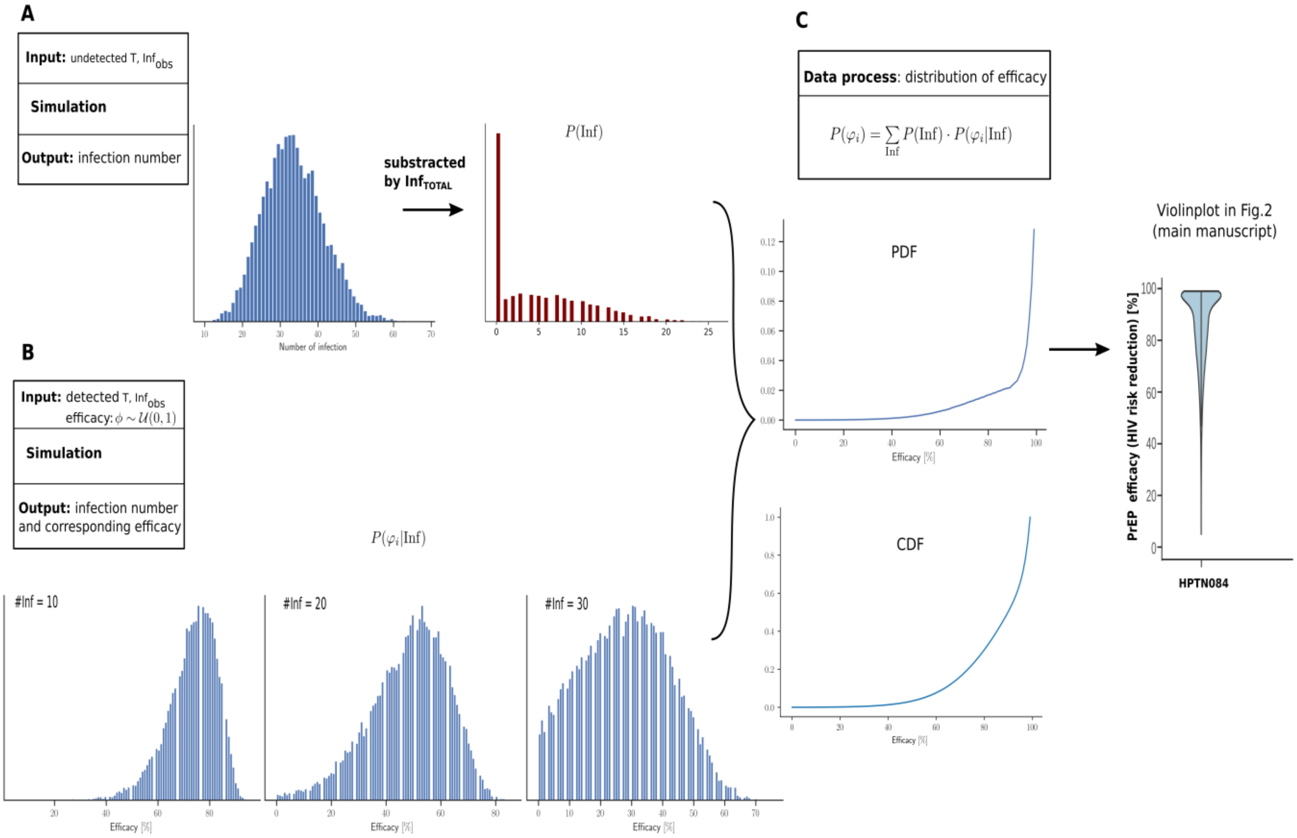

Supplementary Figure S8: Workflow for estimating PrEP efficacy from clinical data. A: Calculation of the number of infections within the 'drug detected' sub-cohort. B: Determination of the efficacy distribution for each generated infection count, where PrEP efficacy is randomly sampled from a uniform distribution in each simulation. C: Derivation of the probability distribution for PrEP efficacy.

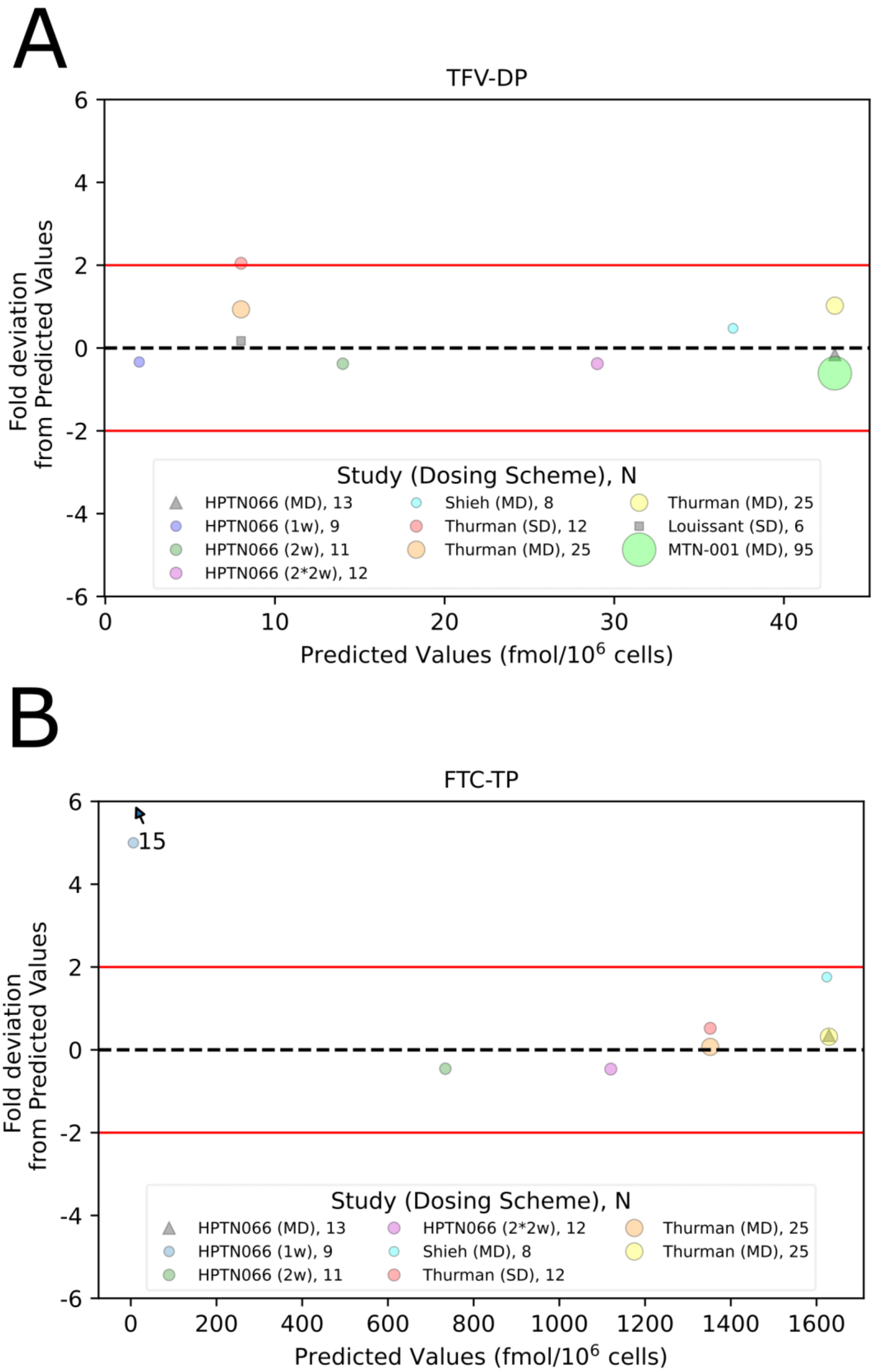

Supplementary Figure S9: Fold deviation of measured- from correspondingly simulated TDF-DP. (A) and FTC-TP (B) concentrations in PBMCs. For each study, the dosing scheme is reported (multiple doses (MD) or single dose (SD), one, two or four tablets per week (1w, 2w, 2*2w)). The number of samples N giving rise to each reported concentration is highlighted and measured mean (or median) concentration markers are scaled proportionally to the inverse sampling error (√N).

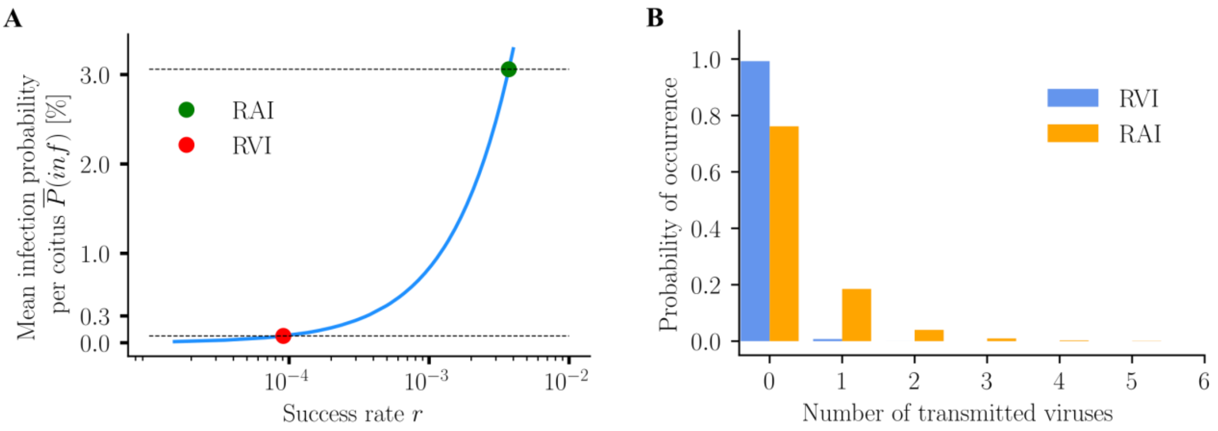

Supplementary Figure S10: Infection probability and inoculum size distribution for receptive vaginal- and anal intercourse (RVI, RAI). A: relation between success rate and the corresponding average infection probability per exposure. Average infection probabilities of RAI and RVI are marked by dots. B: the corresponding inoculum size distribution of RAI and RVI.

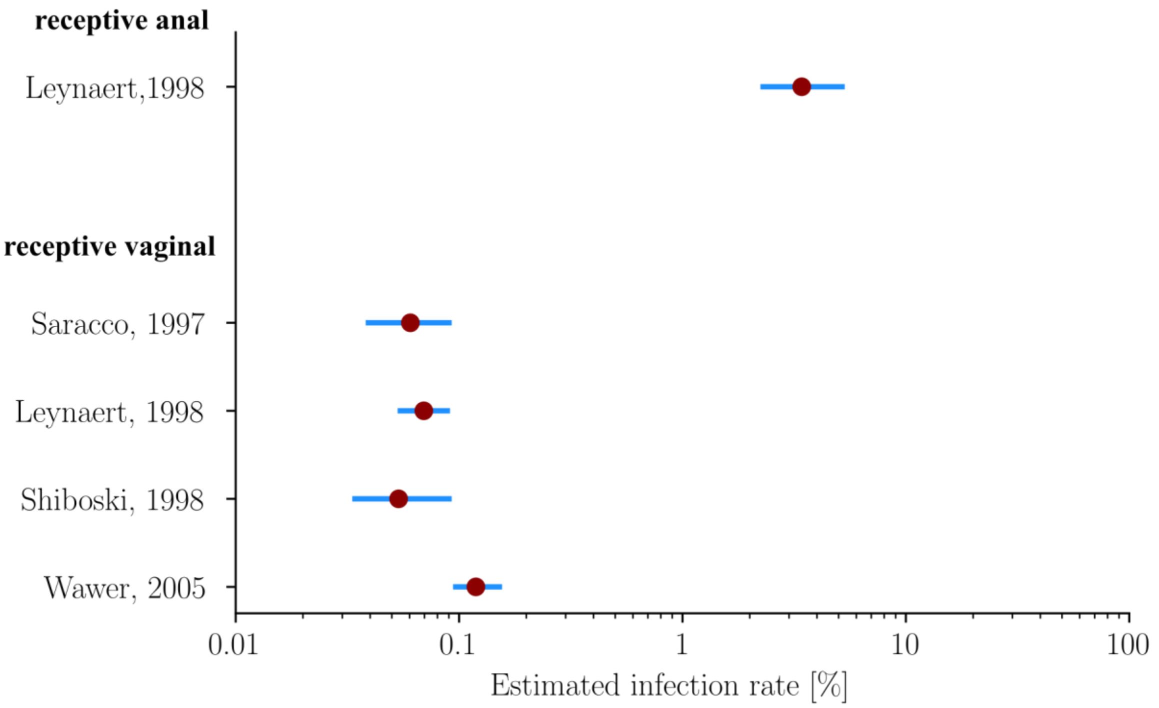

Supplementary Figure S11: Reported infection probabilities for receptive anal intercourse (RAI) and receptive vaginal intercourse (RVI) respectively. Red dots represent means and error bars represent 95% confidence intervals. Data was taken from the literature as indicated (*Online Methods*).

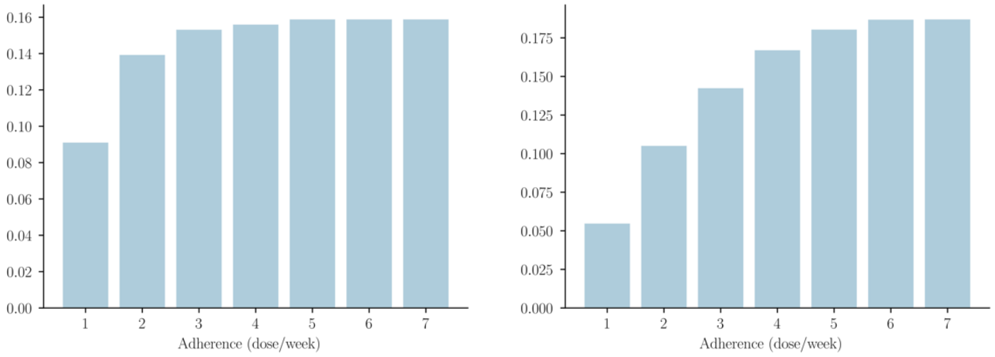

Supplementary Figure S12: Utilized adherence levels for bottom-up simulations in Table 1. Utilized adherence levels to compute PrEP efficacies for different 'bottom-up' modelling scenarios were computed based on the probability that TFV is detectable at a given adherence level. The probability of a certain adherence level in terms of j = 1…7 doses per week was computed as $P_j = \frac{P_j(TFV > LLOQ)}{\sum_{j=1}^{7} P_j(TFV > LLOQ)}$, where P_j(TFV >LLOQ) denotes the average probability that TFV is detectable (above the lower limit of quantification; LLOQ) for j weekly doses, as depicted in Supplementary Figure S1 and S2. A: Utilized adherence levels for LLOQ = 0.001µM (HPTN 084, Partners-PrEP, TDF2 and VOICE study[3,12,22,23]). B: Utilized adherence levels for LLOQ = 0.035µM (FEM-PrEP study[11]).

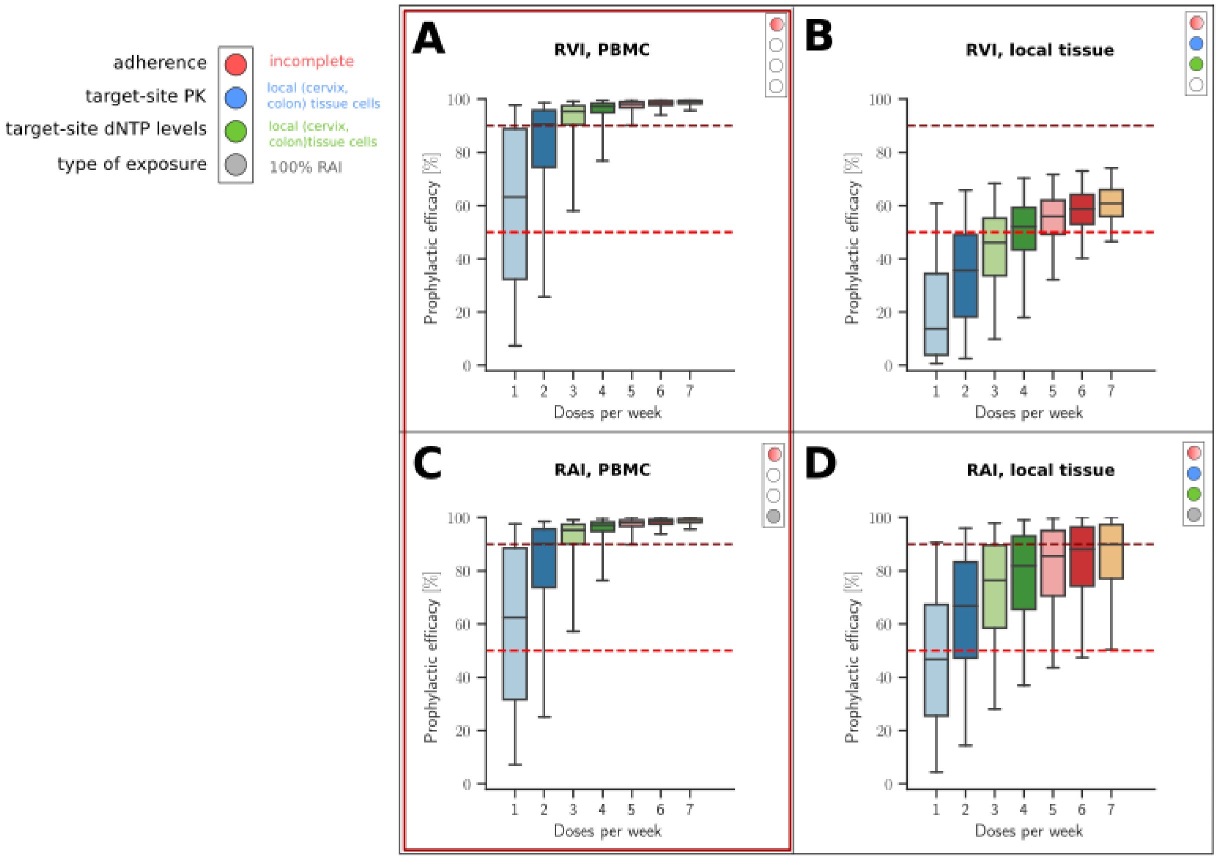

Supplementary Figure S13: Comparison of PrEP efficacy-adherence profiles for receptive vaginal- vs-rectal intercourse. Model-predicted prophylactic efficacy if FTC/TDF was taken once, twice, ..., seven days per week on average after 100% receptive vaginal intercourse (**A-B**) or 100% receptive anal intercourse (**C-D**). In panels **A** and **C**, PBMCs pharmacokinetics were considered the surrogate marker for effect-site concentrations, whereas in panels **B** and **D** local tissue concentrations (vaginal and colorectal respectively) were considered the surrogate marker for effect-site concentrations. N = 1000 virtual patients were sampled. Boxplots show median efficacy and IQR and whiskers extend to 2.5%-97.5% range. 90- and 50% efficacy are highlighted for visual guidance using horizontal dashed black and red lines respectively.

Individual	TFV	TFV-DP	interpretation
E3	*BLQ*	*≈ 100*	*adherence < 1/7 days, no dosing in the last 3 days*
E4	*≈ 110*	*< 100*	*dosing before visit*
E6	***≈ 80***	***>350***	***took the drug***
E7	*≈ 50*	*≈ 200*	*adherence < 1/7 days, no dosing in the last 3 days*
E9	*≈ 400*	*≈ 50*	*dosing before visit*
E12	*≈ 60*	*≈ 170*	*dosing before visit*
E13	*≈ 60*	*≈ 170*	*dosing before visit*
E15	BLQ	<100	*adherence < 1/7 days, no dosing in the last 3 days*
E21	*≈ 400*	*≈ 60*	*dosing before visit*
E28	BLQ	< 100	*adherence < 1/7 days, no dosing in the last 3 days*
E29	BLQ	< 100	*adherence < 1/7 days, no dosing in the last 3 days*
E30	*≈ 100*	*≈ 100*	*dosing before visit*
**E31**	*≈ 30*	**> 350**	***took the drug***
**E33**	BLQ	**>350**	***took the drug in the past, not in the previous 3 days***
E35	BLQ	< 100	*adherence < 1/7 days, no dosing in the last 3 days*
**E36**	*≈ 80*	**> 350**	***took the drug***

Supplementary Table S1: Summary of infected individuals in HPTN084 with detectable drug levels at the time of first HIV positive visit. TFV plasma concentrations are reported in units ng/mL and TFV-DP DBS in units fmol/punch. The limit of quantification was 0.31mg/mL and 31.3 fmol/punch respectively.

Adherence Category	TFV-DP (fmol/punch)	TFV (ng/ml)	Dose/week
Good	>1250	>40	7
Partial	>500-700[★] or >700-1250[†]	>40	4-6
Inconsistent	350-500[★] or 350-700[†]	>40	2-4
Poor	<350	<40	<2

Supplementary Table S2: Adherence-concentration benchmarks for TFV and TFV-DP. BLQ: 0.31 ng/mL for TFV; 31.3 fmol/punch for TFV-DP. [★]After 4 weeks or more on TDF/FTC. [†]After 8 weeks or more on TDF/FTC.

Tissue homogenate:PBMC	TFV-DP	FTC-TP
Colon:PBMC	2.920	0.040
Vaginal:PBMC	0.070	0.060

Supplementary Table S3: Tissue specific conversion factors.
