# Supplementary material for: Model-based predictions of protective HIV pre-exposure prophylaxis adherence levels in cisgender women
Source: Nat Med. 2023 Nov 13;29(11):2753–62. doi: 10.1038/s41591-023-02615-x (PMC10667095; doi:10.1038/s41591-023-02615-x)
Supplement: Supplementary file 2 — Reporting Summary [file 41591_2023_2615_MOESM2_ESM.pdf]

nature portfolio

Corresponding author(s): Max von Kleist

Last updated by author(s): Sep 22, 2023

Reporting Summary

Nature Portfolio wishes to improve the reproducibility of the work that we publish. This form provides structure for consistency and transparency in reporting. For further information on Nature Portfolio policies, see our Editorial Policies and the Editorial Policy Checklist.

Statistics

For all statistical analyses, confirm that the following items are present in the figure legend, table legend, main text, or Methods section.

n/a	Confirmed	
☐	☒	The exact sample size (*n*) for each experimental group/condition, given as a discrete number and unit of measurement
☒	☐	A statement on whether measurements were taken from distinct samples or whether the same sample was measured repeatedly
☐	☒	The statistical test(s) used AND whether they are one- or two-sided<br>*Only common tests should be described solely by name; describe more complex techniques in the Methods section.*
☒	☐	A description of all covariates tested
☐	☒	A description of any assumptions or corrections, such as tests of normality and adjustment for multiple comparisons
☐	☒	A full description of the statistical parameters including central tendency (e.g. means) or other basic estimates (e.g. regression coefficient) AND variation (e.g. standard deviation) or associated estimates of uncertainty (e.g. confidence intervals)
☐	☒	For null hypothesis testing, the test statistic (e.g. *F*, *t*, *r*) with confidence intervals, effect sizes, degrees of freedom and *P* value noted<br>*Give P values as exact values whenever suitable.*
☐	☒	For Bayesian analysis, information on the choice of priors and Markov chain Monte Carlo settings
☒	☐	For hierarchical and complex designs, identification of the appropriate level for tests and full reporting of outcomes
☒	☐	Estimates of effect sizes (e.g. Cohen's *d*, Pearson's *r*), indicating how they were calculated

Our web collection on statistics for biologists contains articles on many of the points above.

Software and code

Policy information about availability of computer code

Data collection	All clinical trial data is publicly available and was extracted from published literature. Data processing is described in Method section. No Software was used for data extraction.
Data analysis	We used custom codes implemented in Python 3.9.2.; the packages used are matplotlib 3.4.1, numpy 1.20.2, pandas 1.2.3, pytorch 1.11.0, scipy 1.6.2. All codes are available at https://github.com/KleistLab/PrEP_TruvadaWomen with DOI: 10.5281/zenodo.8370715

For manuscripts utilizing custom algorithms or software that are central to the research but not yet described in published literature, software must be made available to editors and reviewers. We strongly encourage code deposition in a community repository (e.g. GitHub). See the Nature Portfolio guidelines for submitting code & software for further information.

Data

Policy information about availability of data

All manuscripts must include a data availability statement. This statement should provide the following information, where applicable:

- Accession codes, unique identifiers, or web links for publicly available datasets
- A description of any restrictions on data availability
- For clinical datasets or third party data, please ensure that the statement adheres to our policy

All clinical trial data is publicly available and was extracted from published literature. The pharmacokinetic parameters of virtual patients are provided in Supplementary data.

Research involving human participants, their data, or biological material

Policy information about studies with human participants or human data. See also policy information about sex, gender (identity/presentation), and sexual orientation and race, ethnicity and racism.

Reporting on sex and gender	This research does not involve human research participants, and all data was extracted from published literature. The findings are applicable only to cisgender women.
Reporting on race, ethnicity, or other socially relevant groupings	N/A. The research does not take into account race, ethnicity, or any other socially relevant groupings.
Population characteristics	N/A. Population characteristics is not relevant here.
Recruitment	N/A. Recruitment is not relevant here.
Ethics oversight	N/A. Ethics is not relevant here.

Note that full information on the approval of the study protocol must also be provided in the manuscript.

Field-specific reporting

Please select the one below that is the best fit for your research. If you are not sure, read the appropriate sections before making your selection.

☒ Life sciences ☐ Behavioural & social sciences ☐ Ecological, evolutionary & environmental sciences

For a reference copy of the document with all sections, see nature.com/documents/nr-reporting-summary-flat.pdf

Life sciences study design

All studies must disclose on these points even when the disclosure is negative.

Sample size	The sample size of clinical trial simulations was extracted from published literature. We used data from all major studies evaluating FTC-TDF based PrEP in women. Based on this, the goal of our analysis was to evaluate whether these data sets contained sufficient information.
Data exclusions	No data were excluded from the analyses.
Replication	The reproducibility of the findings was confirmed by (i) repeating the simulations, and (ii) comparing the results obtained through the top-down and bottom-up approaches. We did not carry out clinical studies ourselves. Our analysis was based on five clinical studies, as stated above. The number of stochastic simulations performed in our research is stated in the methods and captions of corresponding figures.
Randomization	We did not form any sub-groups of data except for the 'detected' and 'undetected' sub-cohorts in the clinical trial evaluation, which were based on the data from published literature.
Blinding	N/A. We did not perform clinical studies ourselves (see above).

Reporting for specific materials, systems and methods

We require information from authors about some types of materials, experimental systems and methods used in many studies. Here, indicate whether each material, system or method listed is relevant to your study. If you are not sure if a list item applies to your research, read the appropriate section before selecting a response.

Materials & experimental systems

n/a	Involved in the study	
☒	☐	Antibodies
☒	☐	Eukaryotic cell lines
☒	☐	Palaeontology and archaeology
☒	☐	Animals and other organisms
☐	☒	Clinical data
☒	☐	Dual use research of concern
☒	☐	Plants

Methods

n/a	Involved in the study	
☒	☐	ChIP-seq
☒	☐	Flow cytometry
☒	☐	MRI-based neuroimaging

Clinical data

Policy information about clinical studies

All manuscripts should comply with the ICMJE guidelines for publication of clinical research and a completed CONSORT checklist must be included with all submissions.

Clinical trial registration	We utilize data from clinical trials that are already published.
Study protocol	N/A. We utilize data from clinical trials that are already published.
Data collection	N/A. We utilize data from clinical trials that are already published.
Outcomes	N/A. We utilize data from clinical trials that are already published.
